# Fossil evidence of elytra reduction in ship-timber beetles

**DOI:** 10.1038/s41598-019-41310-1

**Published:** 2019-03-20

**Authors:** Shûhei Yamamoto

**Affiliations:** Integrative Research Center, Field Museum of Natural History, 1400S Lake Shore Drive, Chicago, IL 60605-2496 USA

## Abstract

Beetles (Coleoptera) comprise about one quarter of all described animal species. One of the main contributors to their evolutionary success is the elytra, or hardened forewings, which have protective functions while maintaining their ability to fly. Unlike other beetles, some ship-timber beetles (Lymexylidae) have extremely small elytra and largely exposed functional hindwings. There is little fossil evidence illuminating the evolutionary history of short elytra in lymexylids. Here, I report five well-preserved lymexylid fossils in mid-Cretaceous and Cenozoic ambers from Myanmar (ca. 99 million years ago [Mya]), Russia (ca. 44 Mya), and the Dominican Republic (ca. 16 Mya). Three Cretaceous fossils have strongly reduced, shortened elytra, with unexpected variation in elytral size and shape, whereas very small, modified elytra are found only in much younger Dominican amber. These morphologically diverse extinct lymexylids shed new light on the early origin and evolutionary history of elytra reduction and its diverse variation in the ship-timber beetles. Based on the striking morphological similarities with extant lymexylids, these extinct taxa might have had the same, or similar, ecological, behavioural, and flight modes as the extant ship-timber beetles.

## Introduction

**Figure 1:**
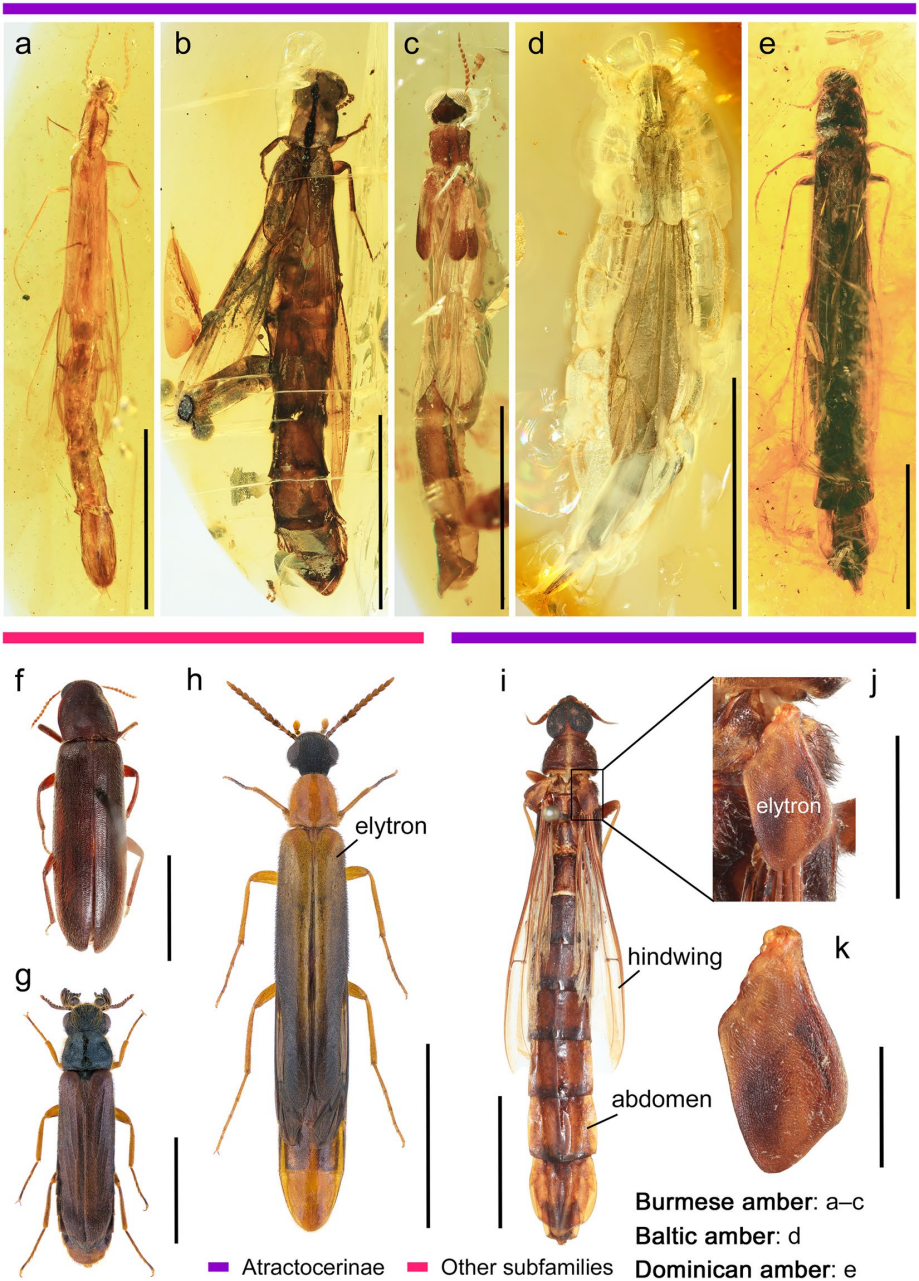
Diverse Lymexylidae beetles, dorsal views. (**a–c)** Atractocerinae from mid-Cretaceous Burmese amber, **d** Atractocerinae from mid-Eocene Baltic amber, **e** Atractocerinae from early Middle Miocene Dominican amber, (**f–k)** extant representatives of Lymexylidae, taken from each subfamily. (**a**) *Vetatractocerus burmiticus* gen. et sp. nov., holotype, FMNHINS-3965988. (**b**) *Raractocetus extinctus* sp. nov., holotype, FMNHINS-3965989. (**c**) *R. fossilis* sp. nov., holotype, FMNHINS-3965990. (**d**) *R. balticus* sp. nov., holotype, FMNHINS-3965991. (**e**) *Atractocerus* sp., FMNHINS-3965992. (**f**) *Melittomma sericeum* (Harris), Melittommatinae, from Indiana, U.S.A., showing fully developed, complete elytra. (**g**) *Hylecoetus dermestoides* (L.), Hylecoetinae, from Derbyshire, U.K., showing well developed, but slightly reduced elytra. (**h**) *Lymexylon navale* (L.), Lymexylinae, from Suffolk, U.K., showing well developed, but moderately reduced elytra. (**i–k**) *A. brasiliensis* Lepeletier & Audinet-Serville, Atractocerinae, from Bluefields, Nicaragua, showing distinctly reduced, minute elytra; (**i**) dorsal habitus, (**j**) enlargement of elytron from (**i**), (**k**) elytron. Photos credits: (**g,h**) by Udo Schmidt, licensed under the Creative Commons Attribution-Share Alike 2.0 Generic license (CC BY-SA 2.0, https://creativecommons.org/licenses/by-sa/2.0/), derived from Wikimedia Commons, (**h**) modified. Scale bars: 3 mm (**a,c,j**), 5 mm (**b,d,e–h**), 1 cm (**i**), 1.5 mm (**k**).

With nearly 400,000 named living species, beetles (Coleoptera) comprise the largest monophyletic order in the Tree of Life, showing astonishing morphological, ecological, and behavioural diversity in nearly all terrestrial and freshwater ecosystems^1–3^. Elytra, which are heavily sclerotised forewings, characterise beetles and cover the dorsal surface of the abdomen. These shield-like structures have various innovative functions in beetles. They protect the hindwings and dorsal abdomen from predators or harsh environmental conditions. Elytra also support desiccation tolerance, minimise the effect of rapid temperature shifts, and even play roles in mimicry or camouflage^4–7^. The strong sclerotisation enabled early beetles to penetrate and adapt to life under bark, like the adults of extant archostematan beetles (Cupedidae and Ommatidae)^8^. Therefore, the presence of elytra makes a major contribution to adaptations to a variety of habitats or micro-environments. Despite these advantages, some beetles possess shortened or reduced elytra. The partial reduction of the elytra, *i*.*e*. brachelytry, is widely scattered across Coleoptera. For example, the rove beetle family Staphylinidae alone contains over 64,000 species and most are brachelytrous. However, the hindwings of rove beetles are hidden under shortened elytra. Although there are some exceptions (see Discussion), this condition of elytra, *i*.*e*. brachelytry with hidden hindwings, are almost universal for the entire Coleoptera. Female trilobite beetles (Lycidae, *Platerodrilus* spp.) are exceptional because they lack trace of elytra^9^. Excluding *Platerodrilus*, the degree of elytral reduction ranges from moderate to almost completely reduced, as seen in the ship-timber beetle subfamily Atractocerinae (Lymexylidae)^7^; the latter have exposed their abdomens and functional hindwings (Fig. 1i,j and Supplementary Fig. 1b).

Lymexylidae is a small, monophyletic beetle family, with only 72 species in 12 genera distributed globally (excluding fossils)^10,11^. Based on several distinct morphological characters (*e*.*g*. highly modified maxillary palpus), lymexylids constitute the sole member of the superfamily Lymexyloidea. This group is so specialised that even a different insect order, Strepsiptera (Stylopidae), was once placed in this superfamily^1^, although this was based on an invalid argument. In some recent studies, Lymexylidae was fully nested within Tenebrionoidea^12–14^ or formed a clade with Tenebrionoidea and Cleroidea^15^. However, the latest, most comprehensive genomic study still recovered it as a distinct superfamily^16^. Lymexylids currently comprise four subfamilies: Atractocerinae (seven genera, one extinct), Hylecoetinae (one genus), Lymexylinae (one genus), and Melittommatinae (five genera, one extinct). Atractocerinae is characterised by exceedingly reduced elytra and is sister to Lymexylinae^10,11^. Direct fossil evidence of the reduced, short elytra in lymexylids has never been provided.

Here, I report five atractocerine beetles in mid-Cretaceous Burmese, mid-Eocene Baltic, and Middle Miocene Dominican ambers. All of the specimens reported here have extremely reduced elytra. The three Burmese amber atractocerines clearly show the reduced elytra, but they also demonstrate some variation in the shape and size of the elytra, ranging from much longer elytra than in the extant taxa to very similar ones. Shorter elytra are also seen in the Baltic amber fossil. Exceedingly minute, shrunken, deformed elytra are represented by only a single occurrence in the much younger Dominican amber. This series of new lymexylid fossils indicates the antiquity of the elytra reduction in the ship-timber beetles and demonstrates various patterns of elytra during the Cretaceous. The extremely reduced lymexylid elytra as in the extant *Atractocerus* are thought to have a post-Cretaceous origin. Although elytra reduction exposing the hindwings and abdomen is seen only in a tiny fraction of the mega-diverse beetles, my discovery broadens our knowledge of the evolution of elytra in Coleoptera.

## Results

### Systematic Palaeontology

Order Coleoptera Linnaeus, 1758

Superfamily Lymexyloidea Fleming, 1821

Family Lymexylidae Fleming, 1821

Subfamily Atractocerinae Laporte, 1840

*Vetatractocerus* gen. nov.

LSID (Life Science Identifier): urn:lsid:zoobank.org:act:EF9F7C08-DA9E-4D63-81D8-D674B74E18EC.

### Type species

*Vetatractocerus burmiticus* sp. nov.

### Etymology

The generic name is a combination of the Latin adjective *vetus* (meaning, ancient) and the genus *Atractocerus*.

### Diagnosis

*Vetatractocerus* is distinguished from all other atractocerines by the following combination of characters: body small (ca. 8.5 mm) and narrow, uniformly pale yellowish brown; head large, rather vertical, slightly wider than pronotum; eyes large, occupying almost the entire frons, eyes nearly contiguous anteriorly; antenna filiform, as long as the head and pronotum combined, each antennomere distinctly elongate; pronotum subparallel sided, moderately produced anteriorly; mesoscutellum small and narrow, occupying about half the width of the elytral base; each elytron reduced, but relatively long and slender, approximately 8.7 times longer than wide, exposing large parts of the abdomen, dorsum lacking marking; and metacoxae remarkably modified, strongly projecting posteriorly.

### Remarks

Among all extant atractocerines, *Vetatractocerus* gen. nov. is morphologically similar to the extant genus *Urtea* Paulus from Greece in having distinctly modified metacoxae, large, contiguous eyes, and anteriorly produced pronotum, but it is easily separated from *Urtea* as follows^11^: body much smaller (16 mm in *Urtea*); eyes smaller, occupying less of the posterior frons; and antennae slenderer and longer. In addition, *Vetatractocerus* gen. nov. can be distinguished from all other Atractocerinae genera as follows^17^: from *Arractocetus*, *Fusicornis*, and *Hymaloxylon* in having large, contiguous eyes; from *Atractocerus* and *Raractocetus* in having slenderer antennae and markedly modified metacoxae; from *Cratoatractocerus* in the absence of M + Cu folk of hindwing, but alternatively having much smaller body (28 mm in *Cratoatractocerus*) and elongate pronotum. In addition, *Vetatractocerus* gen. nov. differs from all extant genera in the presence of longer, slenderer elytra.

**Figure 2:**
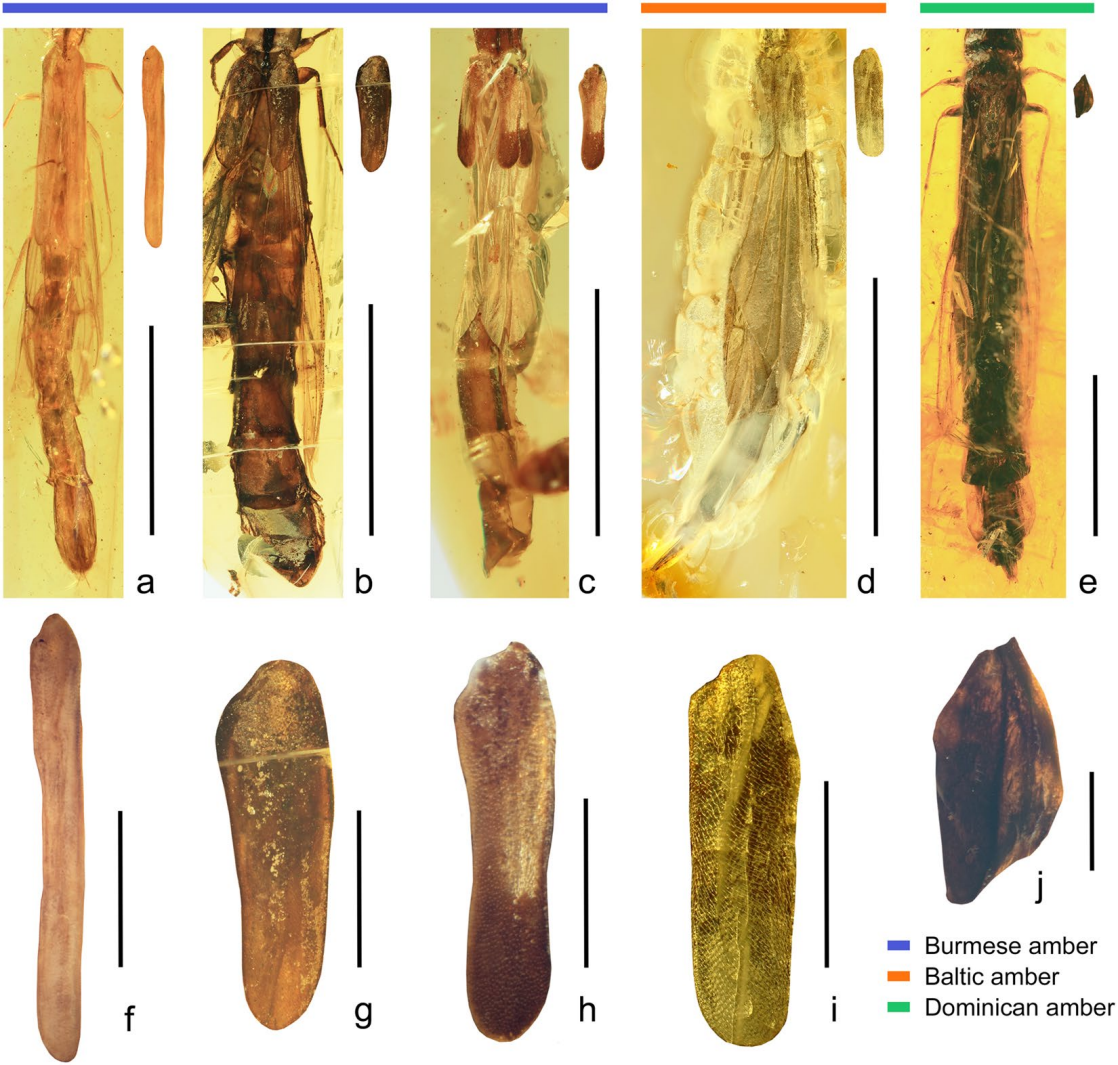
Diversity of reduced elytra in extinct Atractocerinae beetles, dorsal views. (**a–c)** Atractocerinae from mid-Cretaceous Burmese amber, showing short to markedly short elytra, (**d**) Atractocerinae from mid-Eocene Baltic amber, showing very short elytra, **e** Atractocerinae from early Middle Miocene Dominican amber, showing distinctly short and minute elytra. (**a**,**f**) *Vetatractocerus burmiticus* gen. et sp. nov., holotype, FMNHINS-3965988; (**f**) enlargement of elytron. (**b**,**g**) *Raractocetus extinctus* sp. nov., holotype, FMNHINS-3965989; (**g**) enlargement of elytron. (**c**,**h**) *R. fossilis* sp. nov., holotype, FMNHINS-3965990; (**h**) enlargement of elytron. (**d**,**i**) *R. balticus* sp. nov., holotype, FMNHINS-3965991; (**i**) enlargement of elytron. (**e**,**j**) *Atractocerus* sp., FMNHINS-3965992; (**j**) enlargement of elytron. Scale bars: 3 mm (**a,c**), 5 mm (**b,d,e**), 1 mm (**f**,**g**,**i**), 0.5 mm (**h**,**j**).

*Vetatractocerus burmiticus* sp. nov.

(Figures 1a and 2a,f and Supplementary Figs 2a,3)

LSID (Life Science Identifier): urn:lsid:zoobank.org:act:BC26AB2F-E7B3-4A19-B033-5E0574602A17.

### Etymology

The specific epithet is derived from the occurrence of the fossil in Burmese amber known as ‘burmite’.

### Material

Holotype: FMNHINS-3965988 (Supplementary Fig. 2a), a completely preserved female adult. Mid-Cretaceous (earliest Cenomanian) amber [ca. 99 million years ago (Mya)^18^], from the Hukawng Valley, Kachin State, northern Myanmar.

### Diagnosis

As for the genus (see above), with the following minor additions: pronotal disc with blackish and longitudinal complete lines along midline and lateral margins; mesoscutellum with complete, thick, longitudinal blackish line along midline.

### Description

Refer to online Supplemental Note for a complete description.

Genus *Raractocetus* Kurosawa, 1985

### Remarks

This genus was once synonymised^10^, but it was later resurrected^11^. With only two extant species, *Raractocetus* is a small genus in Lymexylidae. The distribution is restricted mainly to the Oriental region, but it is also found in southernmost Australia^11^. The Cretaceous fossils described below are biogeographically consistent with the modern species. By contrast, the occurrence of a *Raractocetus* fossil from Baltic amber is intriguing. The current distribution of Atractocerinae is relatively cosmopolitan, and only a single species of the subfamily is found in Europe, namely *Urtea gracea* Paulus from Greece^11^. Therefore, the discovery of *Raractocetus* in northern Europe is surprising and noteworthy, although this distribution pattern has been known for several beetle groups^19–21^.

*Raractocetus extinctus* sp. nov.

(Figures 1b and 2b,g and Supplementary Figs 2b,4,5).

LSID (Life Science Identifier): urn:lsid:zoobankorg:act: urn:lsid:zoobank.org:act:94130B93-6FFE-4C43-89E6-8B0987C7FEAD.

### Etymology

The specific epithet refers to the Latin adjective *extinct*, highlighting it as a Cretaceous fossil.

### Material

Holotype, FMNHINS-3965989 (Supplementary Fig. 2b), a completely preserved female adult. Mid-Cretaceous (earliest Cenomanian) amber (ca. 99 Mya^18^), from the Hukawng Valley, Kachin State, northern Myanmar.

### Diagnosis

This new species of Atractocerinae can be assigned to the extant genus *Raractocetus* based on the following combination of characters^17^: head rather vertical, moderately wider than pronotum; eyes large, contiguous, occupying almost the entire frons; antenna slender, somewhat fusiform; mesoscutellum relatively narrow, occupying about two thirds the width of the elytral base. *Raractocetus extinctus* sp. nov. can be distinguished from other *Raractocetus* species by the following combination of characters: antennae rather fusiform, thicker; head wide, moderately wider than pronotum; pronotum weakly elongate, anterior margin weakly rounded, pronotal disc with thick deep longitudinal blackish line/groove along the midline; mesoscutellum slightly wider than long, occupying about two thirds the width of the elytral base, dorsum with conspicuous blackish marking along midline; each elytron short, narrowly elongate, approximately 3.2 times longer than wide, gradually narrowing posteriorly, without marking; mesoventrite carinate along midline; and metacoxae rather strongly modified, relatively strongly projecting posteriorly.

### Description

Refer to online Supplemental Note for a complete description.

*Raractocetus fossilis* sp. nov.

(Figures 1c and 2c,h and Supplementary Figs 2c,6,7)

LSID (Life Science Identifier): urn:lsid:zoobank.org:act:ACB32243-DDD8-461B-9D31-FE006252913E.

### Etymology

The specific epithet is derived from the fact that it is a fossil species.

### Material

FMNHINS-3965990 (Supplementary Fig. 2c), a nearly complete adult, but partially damaged, sex undetermined. Mid-Cretaceous (earliest Cenomanian) amber (ca. 99 Mya^18^), from the Hukawng Valley, Kachin State, northern Myanmar.

### Diagnosis

This new species of Atractocerinae can be assigned to the extant genus *Raractocetus* based on the following combination of characters^17^: head rather vertical, slightly wider than pronotum; eyes large, contiguous, occupying almost the entire frons; mesoscutellum relatively small and narrow, occupying about two thirds the width of the elytral base. *Raractocetus fossilis* sp. nov. can be distinguished from other *Raractocetus* species by the following combination of characters: antennae relatively strongly fusiform, slender; head wide, dark brown, slightly wider than pronotum; pronotum subquadrate, very weakly elongate, gradually widened apically, widest and nearly truncate at anterior margin, pronotal disc with thin complete darker line/groove along midline; mesoscutellum as long as wide, occupying about two thirds the width of the elytral base, dorsum with blackish conspicuous marking on base; each elytron slender, approximately 3.9 times longer than wide, narrowest around middle, anterior half with elongate-oval, whitish marking, forming relatively clear pattern; and metacoxae moderately modified, relatively strongly projecting posteriorly.

### Description

Refer to online Supplemental Note for a complete description.

*Raractocetus balticus* sp. nov.

(Figures 1d and 2d,i and Supplementary Figs 2d,8,9).

LSID (Life Science Identifier): urn:lsid:zoobank.org:act:F77CB344-EF73-4EC0-8E6B-AEFDD3AB2D5C.

### Etymology

The specific epithet is derived from the English adjective *baltic* in reference to Baltic amber.

### Material

FMNHINS-3965991 (Supplementary Fig. 2d), a completely preserved female adult. Mid-Eocene Baltic amber (ca. 44 Mya^22^), from Yantarny, Kaliningrad Oblast, Russia.

### Diagnosis

This new species of Atractocerinae can be assigned to the extant genus *Raractocetus* based on the following combination of characters^17^: head rather vertical, slightly wider than pronotum; eyes large, contiguous, occupying almost the entire frons; antenna slender, relatively strongly fusiform; mesoscutellum relatively wide, occupying little more than two thirds the width of the elytral base. *Raractocetus balticus* sp. nov. can be distinguished from other congeners by the following combination of characters: antennae rather strongly fusiform, but slender; head wide, slightly wider than pronotum; pronotum subquadrate, nearly as long as wide, anterior margin weakly rounded, lateral margins gently arcuate, pronotal disc with deep, complete, longitudinal groove along midline (while lacking blackish line); mesoscutellum transverse, occupying little more than two thirds the width of the elytral base, dorsum without markings or maculation; elytron slender, approximately 3.5 times longer than wide, nearly subparallel sided, lacking pattern; and metacoxae moderately modified, relatively strongly projecting posteriorly.

### Description

Refer to online Supplemental Note for a complete description.

### Remarks

*Raractocetus balticus* sp. nov. is the first atractocerine described from Baltic amber.

Genus *Atractocerus* Palisot de Beauvois, 1802

*Atractocerus* sp.

(Figures 1e and 2e,j and Supplementary Figs 2e,10)

### Material

FMNHINS-3965992 (Supplementary Fig. 2e), a complete, relatively well, preserved female adult. Ventral side is largely not visible. Early Middle Miocene amber (ca. 16 Mya^23^), from the Dominican Republic, no further details are available.

### Diagnosis

The fossil can be placed in the extant genus *Atractocerus* using the following diagnostic characters^17^: head clearly narrower than pronotum; large, contiguous eyes; short fusiform antenna; broadened mesoscutellum that occupies more than two thirds the width of the elytral base; and extremely shortened, small elytra. This fossil taxon may be distinguished from other congeners by the following characters: smaller body; strongly fusiform, but distally slender, antennae; minute, modified and elevated elytra; and the structures of the female genitalia. The ventral side is not fully visible. Further comparison with the modern congeners is difficult because of a poor visibility of the ventral side and the ambiguity of the species limit of the extant *Atractocerus*.

### Description

Refer to online Supplemental Note for a complete description.

### Remarks

The distribution of *Atractocerus* in Central and South America (including the Dominican Republic), Africa, Madagascar, India, Sumatra, and northern Australia suggests Gondwanan relictualism^11^. Therefore, the occurrence of *Atractocerus* in Dominican amber is congruent with the modern congeners. Grimaldi & Engel^2^ mentioned a much larger (ca. 29 mm) unnamed *Atractocerus* in Dominican amber, but the elytra were not illustrated.

## Discussion

Three Cretaceous and two Cenozoic beetles unambiguously belong to Lymexylidae, based on their narrowly elongate bodies, cylindrical projecting procoxae, filiform or fusiform, fairly short antennae, highly modified maxillary palp organ, and female genital structures^10,24^. They can be further assigned to Atractocerinae based on the markedly reduced elytra with the exposed hindwings, large bulging eyes, and distinctly modified maxillary palp organ in the female^10^. The diverse atractocerines from Burmese, Baltic, and Dominican ambers reported here represent the first described and named amber inclusions in the subfamily.

**Figure 3:**
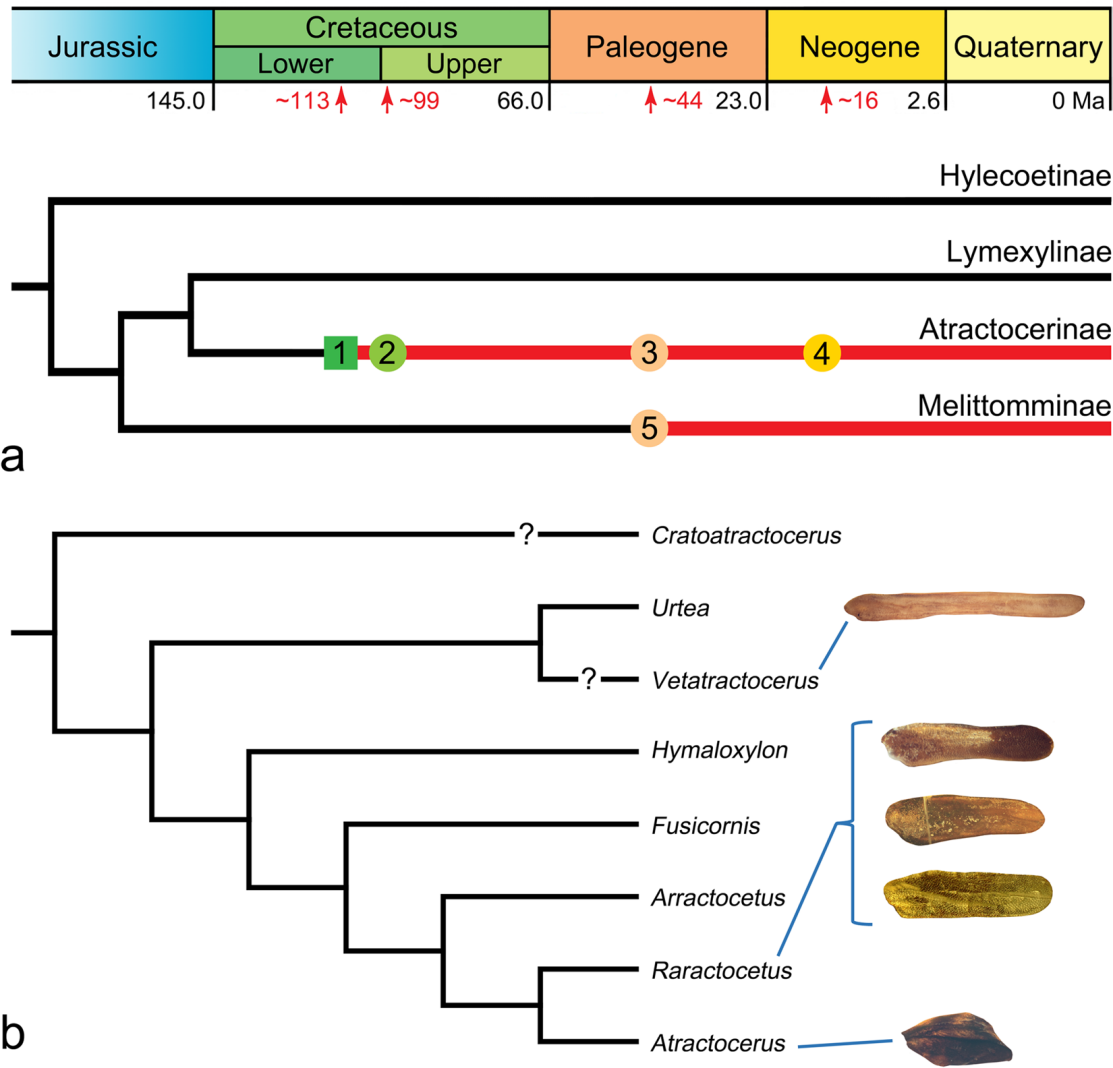
Phylogenetic framework of Lymexylidae and Atractocerinae, based on Wheeler^10^, Paulus^11^, and Wolf-Schwenninger^28^. (**a**) Time-dated cladogram of Lymexylidae, (**b**) hypothesised generic cladogram of Atractocerinae. (**a**) 1: Oldest Atractocerinae from Lower Cretaceous (~113 Mya) Crato Formation, Brazil^28^; 2: Atractocerinae from mid-Cretaceous (ca. 99 Mya) Burmese amber; 3: Atractocerinae from mid-Eocene (ca. 44 Mya) Baltic amber; 4: Atractocerinae from early Middle Miocene (ca. 16 Mya) Dominican amber; 5: oldest Melittommatinae from mid-Eocene Baltic amber^33^. Two older records of Hylecoetinae^29^ and Melittommatinae^31,32^ were omitted due to uncertainty in their systematic positions. Red lines show lineages supported by fossil records. Square, compression fossil. Circles, amber inclusions. (**b**) The elytra of five fossil species reported here are mapped onto the cladogram. *Vetatractocerus* gen. nov. is tentatively placed as a sister taxon to *Urtea* based on the distinctly modified metacoxae, large, contiguous eyes, and anteriorly produced pronotum. *Cratoatractocerus* was considered the most basal member of Atractocerinae based on its hindwing venation, although the general characters resemble those of *Raractocetus*^28^. Since there is significant lack of morphological information on this fossil genus, I tentatively followed the view of Wolf-Schwenninger^28^.

The oldest members of the ship-timber beetles were found in Lower Cretaceous Lebanese amber (~125 Mya)^25^. They are not formally described and therefore no detailed morphological information is available, although they have long, nearly complete elytra^25^. If these fossils are truly lymexylids, this is consistent with the Jurassic-Early Cretaceous origin of the crown group of the family, suggested by previous molecular studies^16,26^. Currently, the earliest definitive fossil of the family is *Cratoatractocerus grimaldii* Wolf-Schwenninger described based on a compression fossil from the Lower Cretaceous Crato Formation (~113 Mya^27^) in Brazil^28^ (Fig. 3a). This fossil species was assigned to the subfamily Atractocerinae based on several characters, including general body shape, large contiguous eyes, two longitudinal ridges on the mesoscutellum, and heavily sclerotised female genitalia^28^. This assignment appears correct, although, unfortunately, the elytra of this fossil taxon were originally missing^28^. Until now, this uncertainty prevented us from elucidating the origin and evolutionary history of elytra reduction in Atractocerinae, since no other fossil lymexylid has been described from the Mesozoic. An undescribed Atractocerinae fossil from Burmese amber has been reported, the authors did not include a figure of the elytra of this fossil^2^. All other family records are confined to Eocene–Miocene (~16 Mya) compression and amber fossils with either long elytra or without sufficient information about them^2,29–33^. My study provides the first documented evidence of the elytral reduction in Lymexylidae, which can now be reliably traced back to the mid-Cretaceous (~earliest Cenomanian, ca. 99 Mya^18^).

In comparison with the mega-diversity of beetles, only a small minority of beetles have reduced elytra with exposed hindwings. Nonetheless, this phenomenon is found repeatedly across the order, indicating multiple independent origins. Because the condition and degree of development of the elytra varies greatly among beetles, a previous study classified the reduced elytra into three morpho-types^7^: 1) elytra reduced along with a reduction in the hindwings, *e*.*g*. Lycidae^34^ and Lampyridae^35^; 2) elytra truncated but completely covering the folded functional hindwings, *e*.*g*. the hyper-diverse Staphylinidae^36^; and 3) reduced elytra exposing (or partially exposing) functional hindwings, *e*.*g*. some Cerambycidae (*i*.*e*. Necydalinae^37^), Ripiphoridae^38^, or Lymexylidae^10^. The third form is the rarest and most interesting elytral type because it apparently does not have a protective function and other merits. Despite these disadvantages, this elytral form is found in unrelated beetle lineages^7^. The origin and role of this type of brachelytry have still not been adequately explored. However, it was suggested that elytra reduction affects the evolution of beetle hindwings by optimising the aerodynamic efficiency or changes in flight mechanics induced by elytra loss^7^. In atractocerine species, extreme cases of reduced elytra are known. Indeed, the elytra are so minute that most of the hindwings and abdomen are exposed dorsally. A remarkable dipteran haltere-like role of minute elytra was revealed by surgically removing the elytra of *Atractocerus brevicornis* (L.)^39,40^. These shortened elytra vibrate during flight and may perform a sensory function essential for stabilising flight, producing the unique flight mode of atractocerines^39,40^. In fact, *A*. *brevicornis* was incapable of steady flight after removing the elytra^39,40^. Based on the minute elytra, my discovery of two new species of *Raractocetus* from Burmese amber suggests that they had a flight mode similar to that of *A*. *brevicornis* during the mid-Cretaceous, as this seems to be a common flight mechanism for the subfamily as a whole.

The fossils described here show a remarkable variation in shape and length of elytra. For example, *Vetatractocerus* gen. nov. differs greatly from the remaining extant and extinct Atractocerinae. With a very slender, unusually long (8.7 times longer than wide) elytron, this new genus is distinct among the atractocerines. More significantly, the length of the elytra and their coverage on the abdomen seems to be intermediate between the subfamily Lymexylinae (Fig. 1h), which is a sister taxon to Atractocerinae, and the other members of Atractocerinae. Nonetheless, the elytra of *Vetatractocerus* gen. nov. are still much shorter than in Lymexylinae, with more than four abdominal tergites exposed dorsally. The presence of a highly modified maxillary palp organ in the female prevents the systematic placement of *Vetatractocerus* gen. nov. within Lymexylinae, but it is definitely placed in Atractocerinae. This new genus might form a sister group with *Urtea* based on the distinctly modified metacoxae, large, contiguous eyes, and anteriorly produced pronotum. These two genera may be nested together at the basal position of Atractocerinae (Fig. 3b), although future studies need to assess the detailed systematic position of *Vetatractocerus* gen. nov. The two Cretaceous *Raractocetus* species have a pair of short, but weakly posteriorly narrowing elytron (Fig. 2b,c,g,h), while one Eocene *Raractocetus* species has a nearly parallel-sided elytron (Fig. 2d,i). Compared with these taxa, one Miocene *Atractocerus* has a shorter (2.1 times longer than wide), distinctly pointed, modified elytron, with an irregularly dorsally raised surface (Fig. 2e,j). Consequently, the modified elytron as seen in extant taxa is considered a derived condition that may have originated by the Middle Miocene, based on my fossil in Dominican amber (ca. 16 Mya).

Of note, there are already some Cretaceous examples of brachelytrous beetles with exposed hindwings. The most notable cases are several wedge-shaped beetles (Ripiphoridae) from mid-Cretaceous French and Burmese ambers^38,41,42^. They clearly exhibit the typical form of reduced elytra with exposed hindwings, although they are much less reduced than the examples presented here. Another example is found in soldier beetles (Cantharidae) from Burmese amber; for example, the genera *Ornatomalthinus*^43^ and *Sanaungulus*^44^ have shortened elytra exposing the hindwings dorsally. These discoveries indicate that reduction in elytral length with exposed hindwings, occurred before the mid-Cretaceous independently in distantly related beetle groups.

Elytra reduction may affect the evolution of hindwing structures^7^. A characteristic feature of recent Atractocerinae is the extremely reduced hindwing venation with a complete lack of transverse folds^28,45,46^. Hindwing shape differs markedly between related brachelytrous and macroelytrous beetles, with the exception of *Atractocerus*, having a wider hindwing than in macroelytrous congeners and having an elongated anal field^28^. Although I could not adequately observe the hindwing shape in the fossils described here, the wing venation patterns were simple and the wings folded lengthwise, as in all extant atractocerine genera; they lack a M + Cu fork basad of the level of the r-m crossvein, clearly differentiated from the Lower Cretaceous *Cratoatractocerus*, and also lack a radial cell along the costal margin of the C + Sc + R vein. The general length of the hindwings is the same as in the extant atractocerines, with at least two free abdominal tergites uncovered by the hindwings. This demonstrates the mid-Cretaceous origin of the modern atractocerine hindwings. Because these atractocerines are unlike examples of beetle taxa inhabiting mesic micro-environments^47–50^, the close morphological similarities with the extant taxa are interesting.

The fossil evidence reported here sheds light on the macroevolution of ship-timber beetles, especially the subfamily Atractocerinae. These morphologically diverse extinct lymexylids highlight the early diversification of elytra reduction in this subfamily. The diverse series of variation in elytral forms reported here also suggest much greater morphological diversity of the elytral shape in the past than seen at present. Interestingly, although only suggestive, the very minute, modified elytra seen in extant *Atractocerus* were found only from younger Dominican amber. The reported fossils here also clearly show several synapomorphic characters of lymexylids, including the modified maxillary palp organ functions as chemoreceptors^51,52^, which represent an autapomorphy of the family (Supplementary Fig. 1c,d)^10^. Another insight is the possible bright colouration of *Vetatractocerus burmiticus* gen. et sp. nov., *Raractocetus extinctus* sp. nov., and *R*. *fossilis* sp. nov. when they were alive, based on their maculation and patterns of the head, pronotum, mesoscutellum, and elytra. It has been hypothesised that the pale species of Atractocerinae, *e*.*g*. *Atractocerus* and *Raractocetus*, with large eyes appear to mimic certain nocturnal wasps (Vespidae and Ichneumonidae)^17,52^. Hence, they may even be considered potential mimics, although more evidence is needed to support this hypothesis. All known lymexylid larvae are wood borers and are believed to form symbiotic associations with ambrosia fungi, *Ascoidea* spp. (Ascomycetes: Saccharomycetales) that grow on the walls of their tunnels^24,52,53^. Considering this, it is possible that these fossil taxa had the same, or similar, ecological lifestyles as the modern ship-timber beetles. Indeed, the *Raractocetus* fossils show striking general morphological similarities to the recent congeners, suggesting they had the same ecological, behavioural, and even flight modes in the mid-Cretaceous.

## Methods

### Materials

The three Cretaceous fossils described herein were obtained from amber from the Hukawng Valley, Kachin State, northern Myanmar (26°21′33.41″N, 96°43′11.88″E). Burmese amber, or burmite, dates to the mid-Cretaceous, currently recognised as the earliest Cenomanian (98.79 ± 0.62 Mya) age based on U–Pb dating of zircon crystals from the volcaniclastic matrix of the amber outcrops^18^. The Baltic amber specimen described here was from Yantarny, Kaliningrad Oblast, westernmost Russia. Although the age of Baltic amber has been the subject of much debate, I follow the mid-Eocene (44.1 ± 1.1 Mya) age from the most recent estimates for the amber-bearing ‘Blue Earth’ formation based on the absolute dating analyses^22^. The Dominican amber fossil comes from the Dominican Republic and further details are unknown. The age of Dominican amber is also somewhat controversial, but I follow the early Middle Miocene (ca. 16 Mya) age^23^. All of the fossil specimens used in this paper are deposited in the insect collection of the Gantz Family Collections Center, Field Museum of Natural History (FMNH), Chicago, IL, USA, with consecutive numbers from FMNHINS-3965988 to FMNHINS-3965992.

### Imaging

Specimens were photographed using a Canon 80D digital camera with a Canon MP-E 65 mm macro lens (F2.8, 1–5 × ) and a Canon MT-24EX macro twin lite as the light source (Figs 1a–e, 2a–e,g–j and Supplementary Figs 3,4,5a–e,g,6a,b,d,7h,8,9). Alternatively, a Dun Ink BK PLUS Lab System mounted on a Canon 6D digital camera, attached with either CF4 or 5 × lens, was used (Fig. 2f and Supplementary Figs 5f, 6c,e,f,7a–g,10). All images were later stacked using the auto-montage software Combine ZM or Helicon Focus 5.3. Figures were edited and assembled with Adobe Photoshop^®^ Elements 15. Specimens were studied under a Leica MZ16 stereomicroscope.

### Classification

I follow the classifications of Kurosawa^17^ and Paulus^11^.

### Morphological terminology

The morphological terminology generally follows Kurosawa^17^ and Wheeler^10^ for general body parts, and Wolf-Schwenninger^28^ for wing venation. Abbreviations of the hindwing venation are as follows: C, costa; Cu, cubitus; M, median vein; R, radius; r-m, a cross vein that connects M and Cu; Sc, subcosta.

### Nomenclatural acts

This published work and the nomenclatural acts it contains have been registered in ZooBank, the proposed online registration system for the International Code of Zoological Nomenclature (ICZN). The ZooBank LSIDs (Life Science Identifiers) can be resolved and the associated information viewed through any standard web browser by appending the LSID to the prefix ‘http://zoobank.org/’. The LSIDs for this publication are: urn:lsid:zoobank.org:pub:256304D4-479E-44B5-A14D-893D9E898479; urn:lsid:zoobank.org:act:EF9F7C08-DA9E-4D63-81D8-D674B74E18EC; urn:lsid:zoobank.org:act:BC26AB2F-E7B3-4A19-B033-5E0574602A17; urn:lsid:zoobank.org:act:94130B93-6FFE-4C43-89E6-8B0987C7FEAD; urn:lsid:zoobank.org:act:ACB32243-DDD8-461B-9D31-FE006252913E;

urn:lsid:zoobank.org:act:F77CB344-EF73-4EC0-8E6B-AEFDD3AB2D5C.

## Supplementary information

Supplemental information

## Data Availability

All fossil materials are deposited in the FMNH. The data supporting the study findings are provided in both the paper and Supplementary Information. Higher-resolution figures have been deposited in the figshare database (10.6084/m9.figshare.7800830).
