# Supplementary material for: Fossil evidence of elytra reduction in ship-timber beetles
Source: Sci Rep. 2019 Mar 20;9:4938. doi: 10.1038/s41598-019-41310-1 (PMC6426864; doi:10.1038/s41598-019-41310-1)
Supplement: Supplementary file 1 — Supplemental information [file 41598_2019_41310_MOESM1_ESM.pdf]

SUPPLEMENTARY INFORMATION

Title: Fossil evidence of elytra reduction in ship-timber beetles

Author: Shûhei Yamamoto

Supplemental Note

Systematic Palaeontology.

Order Coleoptera Linnaeus, 1758

Superfamily Lymexyloidea Fleming, 1821

Family Lymexylidae Fleming, 1821

Subfamily Atractocerinae Laporte, 1840

Vetatractocerus gen. nov.

LSID (Life Science Identifier): urn:lsid:zoobank.org:act:EF9F7C08-DA9E-4D63-81D8-D674B74E18EC.

Type species. *Vetatractocerus burmiticus* sp. nov.

Etymology. The generic name is a combination of the Latin adjective *vetus* (meaning, ancient) and the genus *Atractocerus*.

Diagnosis. *Vetatractocerus* is distinguished from all other atractocerines by the following combination of characters: body small (ca. 8.5 mm) and narrow, uniformly pale yellowish brown; head large, rather vertical, slightly wider than pronotum; eyes large, occupying almost the entire frons, eyes nearly contiguous anteriorly; antenna filiform, as long as the head and pronotum combined, each antennomere distinctly elongate; pronotum subparallel sided, moderately produced anteriorly; mesoscutellum small and narrow, occupying about half the width of the elytral base; each elytron reduced, but relatively long and slender, approximately 8.7 times longer than wide, exposing larger parts of the abdomen, dorsum lacking marking; and metacoxae remarkably modified, strongly projecting posteriorly.

Remarks. Among all extant atractocerines, *Vetatractocerus* gen. nov. is morphologically similar to the extant genus *Urtea* Paulus from Greece in having distinctly modified metacoxae, large, contiguous eyes, and anteriorly produced pronotum, but it is easily separated from *Urtea* as follows[11]: body much smaller (16 mm in *Urtea*); eyes smaller,

occupying less of the posterior frons; and antennae slenderer and longer. In addition, *Vetatractocerus* gen. nov. can be distinguished from all other Atractocerinae genera as follows[17]: from *Arractocetus*, *Fusicornis*, and *Hymaloxylon* in having large, contiguous eyes; from *Atractocerus* and *Raractocetus* in having slenderer antennae and markedly modified metacoxae; from *Cratoatractocerus* in the absence of M+Cu folk of hindwing, but alternatively having much smaller body (28 mm in *Cratoatractocerus*) and elongate pronotum. In addition, *Vetatractocerus* gen. nov. differs from all extant genera in the presence of longer, slenderer elytra.

Vetatractocerus burmiticus sp. nov.

(Figs. 1a, 2a, f and Supplementary Figs. 2a, 3)

LSID (Life Science Identifier): urn:lsid:zoobank.org:act:BC26AB2F-E7B3-4A19-B033-5E0574602A17.

Etymology. The specific epithet is derived from the occurrence of the fossil in Burmese amber known as 'burmite'.

Material. Holotype: FMNHINS-3965988 (Supplementary Fig. 2a), a completely preserved female adult. Mid-Cretaceous (earliest Cenomanian) amber [ca. 99 million years ago (Mya)[18]], from the Hukawng Valley, Kachin State, northern Myanmar.

Diagnosis. As for the genus (see above), with the following minor additions: pronotal disc with blackish and longitudinal complete lines along midline and lateral margins; mesoscutellum with complete, thick, longitudinal blackish line along midline.

Description. Female. Body (Fig. 1a and Supplementary Fig. 3a) small, 8.54 mm long, elongate, distinctly narrowly subparallel sided. Colour uniformly pale yellowish brown; pronotum with complete, longitudinal blackish lines along midline and lateral margins (Fig. 1a and Supplementary Fig. 3c, e); mesoscutellum with complete, thick, longitudinal blackish line along midline (Supplementary Fig. 3c). Surface leathery; dorsal surface covered with fine setose punctures. Head (Supplementary Fig. 3b, c, e) large, 0.41 mm long and 0.56 mm wide, broadly oval, rather vertical, slightly wider than pronotum; basal part of head narrowed behind eyes, forming loose neck. Eyes (Supplementary Fig. 3b, c, e) large, conspicuous, nearly contiguous anteriorly, occupying extensive parts of head surface. Antennae (Supplementary Fig. 3f) 11-segmented, long, filiform, nearly parallel sided; each antennomere distinctly elongate. Frontoclypeal suture absent. Mandibles minute,

inconspicuous. Maxillary palpus 4-segmented; terminal palpomere highly modified into palp organ (Supplementary Fig. 3b, e). Pronotum (Supplementary Fig. 3c, e) small, 0.94 mm long and 0.52 mm wide (length/width = 1.81), subconical in anterior half, with somewhat strongly produced anterior margin; pronotal disc with blackish and fully developed longitudinal lines on median and lateral margins (Supplementary Fig. 3c, e). Mesoscutellum (Supplementary Fig. 3c) small and narrow, occupying about half width of elytral base; lateral areas depressed. Elytra (Figs. 1a, 2a, f) brachelytrous, but relatively long, exposing larger part of abdomen; elytral base rather narrowly strongly produced anteriorly; each elytron markedly long and slender, approximately 8.7 times longer than wide (2.78 mm long, 0.32 mm wide); dorsum simple, lacking markings or maculation. Metaventrite (Supplementary Fig. 3a, d) distinctly elongate. Hindwing (Figs. 1a, 2a) exposed, not folded beneath elytra; marginal C+Sc+R vein well developed, without radial cell; r-m crossvein (Figs. 1a, 2a) present, conspicuous; M+Cu fork absent. Legs markedly slender, thin, fragile; procoxae (Supplementary Fig. 3d) long, cylindrical; protibiae (Supplementary Fig. 3e) without tibial terminal spur; protarsi (Supplementary Fig. 3e) 5-segmented, long, as long as protibiae; mesocoxae (Supplementary Fig. 3d), very long, clavate; mesotibia (Supplementary Fig. 3a, d) thin, bar-like, with single short spur; mesotarsi 5-segmented, long, moderately longer than mesotibiae; metacoxae (Supplementary Fig. 3a, d) markedly modified, extremely strongly produced posteriorly; metatibia (Supplementary Fig. 3a) thin, bar-like, as long as mesotibiae, with single short spur; metatarsi 5-segmented, long, moderately longer than metatibiae. Abdomen (Figs. 1a, 2a and Supplementary Fig. 3a, g) very slender, cylindrical, moderately flattened, with at least six free ventrites. Female genitalia (Supplementary Fig. 3g) partially exposed; distal part of coxite slender, narrow; stylus long, very slender, gradually weakly thickened apically.

Genus *Raractocetus* Kurosawa, 1985

Remarks. This genus was once synonymised[10], but it was later resurrected[11]. With only two extant species, *Raractocetus* is a small genus in Lymexylidae. The distribution is restricted mainly to the Oriental region, but it is also found in southernmost Australia[11]. The Cretaceous fossils described below are biogeographically consistent with the modern species. By contrast, the occurrence of a *Raractocetus* fossil from Baltic amber is intriguing. The current distribution of Atractocerinae is relatively cosmopolitan, and only a single

species of the subfamily is found in Europe, namely *Urtea gracea* Paulus from Greece [11]. Therefore, the discovery of the subfamily in northern Europe is surprising and noteworthy.

Raractocetus extinctus sp. nov.

(Figs. 1b, 2b, g and Supplementary Figs. 2b, 4, 5)

LSID (Life Science Identifier): urn:lsid:zoobankorg:act:
urn:lsid:zoobank.org:act:94130B93-6FFE-4C43-89E6-8B0987C7FEAD.

Etymology. The specific epithet refers to the Latin adjective *extinct*, highlighting it as a Cretaceous fossil.

Material. Holotype, FMNHINS-3965989 (Supplementary Fig. 2b), a completely preserved female adult. Mid-Cretaceous (earliest Cenomanian) amber (ca. 99 Mya[18]), from the Hukawng Valley, Kachin State, northern Myanmar.

Diagnosis. This new species of Atractocerinae can be assigned to the extant genus *Raractocetus* based on the following combination of characters[17]: head rather vertical, moderately wider than pronotum; eyes large, contiguous, occupying almost the entire frons; antenna slender, somewhat fusiform; mesoscutellum relatively narrow, occupying about two thirds the width of the elytral base. *Raractocetus extinctus* sp. nov. can be distinguished from other *Raractocetus* species by the following combination of characters: antennae rather fusiform, thicker; head wide, moderately wider than pronotum; pronotum weakly elongate, anterior margin weakly rounded, dorsum with thick deep longitudinal blackish line/groove along the midline; mesoscutellum slightly wider than long, occupying about two thirds the width of the elytral base, pronotal disc with conspicuous blackish marking along midline; each elytron short, narrowly elongate, approximately 3.2 times longer than wide, gradually narrowing posteriorly, without marking; mesoventrite carinate along midline; and metacoxae rather strongly modified, relatively strongly projecting posteriorly.

Description. Female. Body (Fig. 1b and Supplementary Fig. 4a) small, 13.44 mm long, elongate, robust, narrowly subparallel sided, moderately dorsoventrally flattened. Colour uniformly pale to dark yellowish brown; head, pronotum, and mesoscutellum, with thick, blackish, and consecutive midline (Fig. 1b and Supplementary Figs. 4b, d, 5a); pronotum additionally margined laterally with blackish lines (Supplementary Fig. 4b, d). Surface leathery, covered with fine setose punctures. Head (Supplementary Fig. 4b–d) large and wide, 0.98 mm long and 1.37 mm wide, broadly oval, rather vertical, moderately wider than pronotum; basal part of head narrowed behind eyes, forming very loose neck. Eyes

(Supplementary Fig. 3b–d) large, conspicuous, nearly contiguous, occupying extensive parts of head surface. Antenna inserted under lateral, supra-antennal ridge. Antennae (Supplementary Fig. 4b, d) 11-segmented, short, more or less fusiform, thick. Frontoclypeal suture absent. Labrum small, transverse. Mandibles small, broad; apex bidentate. Maxillary palpus 4-segmented; terminal palpomere highly modified into weakly developed palp organ (Supplementary Fig. 4c). Labial palpomere 3 (Supplementary Fig. 4c) flattened, elongate oval. Pronotum (Supplementary Fig. 4b, d) small, 1.61 mm long and 1.14 mm wide, weakly elongate (length/width = 1.41), subparallel sided, with weakly rounded anterior margin; dorsum with thick and deep longitudinal blackish line along midline, of which moderately grooved in posterior half. Mesoscutellum (Supplementary Fig. 5a) relatively narrow, slightly wider than long, occupying about two thirds width of elytral base; lateral areas depressed. Elytra (Figs. 1b, 2b, g and Supplementary Fig. 5a) brachelytrous, gradually narrowing posteriorly, exposing most part of abdomen; each elytron narrowly elongate, slender, approximately 3.2 times longer than wide (2.38 mm long, 0.74 mm wide); lateral margins moderately arcuate; dorsum simple, lacking markings or maculation. Mesoventrite (Supplementary Fig. 5e) rather strongly carinate along midline. Metaventrite (Supplementary Fig. 5e) distinctly elongate. Hindwing (Figs. 1b, 2b and Supplementary Figs. 4a, 5f) exposed, not folded beneath elytra; marginal C+Sc+R vein well developed, without radial cell; r-m crossvein (Supplementary Fig. 5f) present, relatively thick; M+Cu fork absent. Legs slender and thin; procoxae long, robust, cylindrical; protibiae (Supplementary Fig. 4b) without tibial terminal spur; protarsi (Supplementary Fig. 4b) 5-segmented, long, as long as protibiae; mesocoxal cavities narrowly separated; mesocoxae (Supplementary Fig. 4c) long, cylindrical; mesotibia (Supplementary Fig. 5g) bar-like, with single short spur; mesotarsi (Supplementary Fig. 5g) 5-segmented, long, moderately longer than mesotibiae; metacoxae (Supplementary Figs. 4a, 5b, e) relatively strongly modified, strongly produced posteriorly; metatibia (Supplementary Fig. 5b) with single short spur; metatarsi 5-segmented, distinctly long, much longer than metatibiae. Abdomen (Figs. 1b, 2b and Supplementary Figs. 4a, 5b–f) slender, cylindrical, moderately flattened, with six free ventrites. Female genitalia (Supplementary Fig. 5c, d) barely exposed; distal part of stylus, slender, gradually weakly widened apically.

Remarks. Among the members of *Raractocetus*, *R. extinctus* sp. nov. can be comparable with the other two extinct species in the presence of relatively strongly projecting metacoxae. However, *R. extinctus* sp. nov. can be readily separated from them as follows:

from *R. fossilis* sp. nov. in having much larger body (7.6 mm in *R. fossilis* sp. nov.), wider head, thicker antennae, paler head, unicolorous elytra, and distinct longitudinal blackish line in the fore body; from *R. balticus* sp. nov. in having wider head, thicker antennae, parallel-sided pronotum, narrower mesoscutellum, carinate mosoventrite, and longitudinal blackish line in the fore body.

Raractocetus fossilis sp. nov.

(Figs. 1c, 2c, h and Supplementary Figs. 2c, 6, 7)

LSID (Life Science Identifier): urn:lsid:zoobank.org:act:ACB32243-DDD8-461B-9D31-FE006252913E.

Etymology. The specific epithet is derived from the fact that it is a fossil species.

Material. FMNHINS-3965990 (Supplementary Fig. 2c), a nearly complete adult, but partially damaged, sex undetermined. Mid-Cretaceous (earliest Cenomanian) amber (ca. 99 Mya[18]), from the Hukawng Valley, Kachin State, northern Myanmar.

Diagnosis. This new species of Atractocerinae can be assigned to the extant genus *Raractocetus* based on the following combination of characters[17]: head rather vertical, slightly wider than pronotum; eyes large, contiguous, occupying almost the entire frons; mesoscutellum relatively small and narrow, occupying about two thirds the width of the elytral base. *Raractocetus fossilis* sp. nov. can be distinguished from other *Raractocetus* species by the following combination of characters: antennae relatively strongly fusiform, slender; head wide, dark brown, slightly wider than pronotum; pronotum subquadrate, very weakly elongate, gradually widened apically, widest and nearly truncate at anterior margin, pronotal disc with thin complete darker line/groove along midline; mesoscutellum as long as wide, occupying about two thirds the width of the elytral base, dorsum with blackish conspicuous marking on base; each elytron slender, approximately 3.9 times longer than wide, narrowest around middle, anterior half with elongate-oval, whitish marking, forming relatively clear pattern; and metacoxae moderately modified, relatively strongly projecting posteriorly.

Description. Sex undetermined. Body (Fig. 1c and Supplementary Fig. 6a) small, 7.60 mm long, elongate, narrowly subparallel sided. Colour uniformly pale brown, but head darker; pronotum with darker, shallow, and complete groove along midline (Fig. 1c and Supplementary Fig. 6c); elytron each with elongate-oval paler maculation near outer margin in anterior half (Figs. 1c, 2c, h and Supplementary Fig. 6c). Surface leathery,

covered with fine setose punctures. Head (Supplementary Fig. 6c–e) large, 0.49 mm long and 0.72 mm wide, broadly oval, rather vertical, slightly wider than pronotum; basal part of head moderately narrowed behind eyes. Eyes (Supplementary Fig. 6c–f) large, conspicuous, nearly contiguous, occupying extensive parts of head surface. Antenna inserted under lateral, supra-antennal ridge. Antennae (Supplementary Fig. 6d, e) 11-segmented, short, more or less fusiform, slender. Frontoclypeal suture absent. Labrum small, transverse. Maxillary palpus 4-segmented; terminal palpomere highly modified into moderately developed palp organ (Supplementary Fig. 6d, f). Labial palpomere 3 elongate oval. Pronotum (Supplementary Fig. 6c, d) small, 0.70 mm long and 0.65 mm wide, subquadrate, only weakly elongate (length/width = 1.10), gradually widened apically, with nearly truncate anterior margin and weakly medially produced posterior margin; pronotal disc with thin, but complete, longitudinal darker groove along midline. Mesoscutellum (Supplementary Fig. 6c) relatively small and narrow, as long as wide, occupying about two thirds width of elytral base; dorsum strongly carinate, lateral areas well depressed, with darker marking on median area. Elytra (Figs. 1c, 2c, h, and Supplementary Figs. 6c, d, 7c, g) brachelytrous, slender, nearly subparallel sided, narrowest in middle, exposing most part of abdomen; each elytron narrowly elongate, slender, approximately 3.9 times longer than wide (1.18 mm long, 0.30 mm wide); dorsum of each elytron with large, elongate-oval, whitish marking in anterior half. Metaventrite (Supplementary Figs. 6a, 7a, g) distinctly elongate. Metepisternum (Supplementary Fig. 7g) and metepimeron (Supplementary Fig. 7g) long, narrow, narrowing posteriorly. Hindwing (Figs. 1c, 2c and Supplementary Figs. 6b, 7d) exposed, not folded beneath elytra; marginal C+Sc+R vein well developed, without radial cell; r-m crossvein (Supplementary Fig. 7d) present, inconspicuous; M+Cu fork absent. Legs slender and thin; procoxae (Supplementary Figs. 6d, 7a) long, robust, clavate, flattened probably due to preservation process; protibiae (Supplementary Fig. 7e) without tibial terminal spur; protarsi (Supplementary Fig. 7e) 5-segmented, long, slightly longer than protibiae; mesocoxae (Supplementary Fig. 6d) long, clavate, flattened probably due to preservation process; mesotibia (Supplementary Fig. 6b, d) bar-like, with single short, thick spur; mesotarsi (Supplementary Fig. 7g) 5-segmented, long, slightly longer than mesotibiae; metacoxae (Supplementary Figs. 6a, 7a, d) moderately modified, strongly produced posteriorly; metatibia with single short, thick spur; metatarsi (Supplementary Fig. 7f) 5-segmented, distinctly long, much longer than metatibiae. Abdomen (Figs. 1c, 2c and Supplementary Fig. 6a, b) slender, cylindrical, posteriorly partially broken, with six free ventrites. Genitalia not preserved.

Remarks. Among the members of *Raractocetus, R. fossilis* sp. nov. can be comparable with the other two extinct species in the presence of relatively strongly projecting metacoxae. However, *R. fossilis* sp. nov. can be readily separated from them as follows: from *R. extinctus* sp. nov. in having much smaller body (13.4 mm in *R. extinctus* sp. nov.), narrower head, slender antennae, darker head, and distinct elytral maculation, lacking distinct longitudinal blackish line in the fore body; from *R. balticus* sp. nov. by the general shapes of pronotum and elytra, narrower mesoscutellum, darker head, and distinct elytral maculation.

Raractocetus balticus sp. nov.

(Figs. 1d, 2d, i and Supplementary Figs. 2d, 8, 9)

LSID (Life Science Identifier): urn:lsid:zoobank.org:act:F77CB344-EF73-4EC0-8E6B-AEFDD3AB2D5C.

Etymology. The specific epithet is derived from the English adjective *baltic* in reference to Baltic amber.

Material. FMNHINS-3965991 (Supplementary Fig. 2d), a completely preserved female adult. Mid-Eocene Baltic amber (ca. 44 Mya[22]), from Yantarny, Kaliningrad Oblast, Russia.

Diagnosis. This new species of Atractocerinae can be assigned to the extant genus *Raractocetus* based on the following combination of characters[17]: head rather vertical, slightly wider than pronotum; eyes large, contiguous, occupying almost the entire frons; antenna slender, relatively strongly fusiform; mesoscutellum relatively wide, occupying little more than two thirds the width of the elytral base. *Raractocetus balticus* sp. nov. can be distinguished from other congeners by the following combination of characters: antennae rather strongly fusiform, but slender; head wide, slightly wider than pronotum; pronotum subquadrate, nearly as long as wide, anterior margin weakly rounded, lateral margins gently arcuate, pronotal disc with deep, complete, longitudinal groove along midline (while lacking blackish line); mesoscutellum transverse, occupying little more than two thirds the width of the elytral base, dorsum without markings or maculation; elytron slender, approximately 3.5 times longer than wide, nearly subparallel sided, lacking pattern; and metacoxae moderately modified, relatively strongly projecting posteriorly.

Description. Female. Body (Fig. 1d and Supplementary Fig. 9a) small, 11.14 mm long excluding exposed female genitalia, elongate, robust, narrowly subparallel sided. Colour uniformly pale yellowish brown, partially covered with whitish micro-bubbles, especially

for ventral side; head, pronotum, and mesoscutellum, without markings or maculation. Surface leathery, covered with fine setose punctures, each seta relatively long. Head (Supplementary Fig. 8) large, 0.58 mm long and 0.92 mm wide, broadly oval, rather vertical, slightly wider than pronotum; basal part of head moderately narrowed behind eyes. Eyes (Supplementary Fig. 8) large, conspicuous, nearly contiguous, occupying extensive parts of head surface. Antenna inserted under lateral, supra-antennal ridge. Antennae (Supplementary Fig. 8e) 11-segmented, short, relatively strongly fusiform, slender. Maxillary palpus 4-segmented; terminal palpomere highly modified into moderately developed palp organ (Supplementary Fig. 8d, e). Labial palpomere 3 flattened, securiform. Pronotum (Supplementary Fig. 8a, b) small, 0.88 mm long and 0.81 mm wide, subquadrate, nearly as long as wide (length/width = 1.09); lateral margins gently arcuate, with moderately rounded anterior and posterior margins; pronotal disc with deep, complete, and longitudinal groove along midline (while lacking blackish line). Mesoscutellum (Supplementary Fig. 8a) relatively large, transverse, occupying more than two thirds width of elytral base; dorsum moderately carinate, lateral areas weakly depressed, lacking darker maculation on median area. Elytra (Figs. 1d, 2d and Supplementary Fig. 8a) brachelytrous, nearly subparallel sided, exposing most part of abdomen; each elytron narrowly elongate, slender, approximately 3.5 times longer than wide (2.09 mm long, 0.59 mm wide); dorsum rather flattened, lacking markings or maculation. Mesoventrite (Supplementary Fig. 9f) smooth, lacking carina along midline. Metaventrite (Supplementary Fig. 9a, b) distinctly elongate. Hindwing (Figs. 1a, 2d and Supplementary Fig. 9g) exposed, not folded beneath elytra; marginal C+Sc+R vein (Supplementary Fig. 9g) well developed, without radial cell; r-m crossvein (Supplementary Fig. 9g) present, conspicuous; M+Cu fork absent. Legs slender and thin; procoxae (Supplementary Fig. 9a, f) thick, robust, more or less cylindrical; protibiae (Supplementary Fig. 9f) without tibial terminal spur; protarsi (Supplementary Fig. 9f) long, as long as protibiae, segmentation not well visible; mesocoxal cavities nearly contiguous; mesocoxae (Supplementary Fig. 9f), long, cylindrical; mesotibia (Supplementary Fig. 9b, c) bar-like, with single short spur; mesotarsi (Supplementary Fig. 9b, c) 5-segmented, long, slightly longer than mesotibiae; metacoxae (Supplementary Fig. 9a, b) moderately modified, strongly produced posteriorly; metatibia (Supplementary Fig. 9d) with single short spur; metatarsi (Supplementary Fig. 9d) distinctly long, much longer than metatibiae, segmentation not well visible. Abdomen (Figs. 1d, 2d and Supplementary Fig. 9a, b, d, e) slender, cylindrical, moderately flattened, with at least five free ventrites. Female genitalia (Fig. 2d and Supplementary Fig. 9a, e) largely exposed, well sclerotised;

coxites slender, narrowly elongate; stylus narrowly elongate, slender, gradually widened apically.

Remarks. *Raractocetus balticus* sp. nov. is the first atractocerine described from Baltic amber. Among the members of *Raractocetus*, *R. balticus* sp. nov. can be comparable with the other two extinct species in the presence of relatively strongly projecting metacoxae. However, *R. balticus* sp. nov. can be readily separated from them as follows: from *R. extinctus* sp. nov. in having narrower head, slenderer antennae, laterally arcuate margins of pronotum, wider mesoscutellum, non-carinate mesoventrite, and the absence of longitudinal blackish line in the fore body; from *R. fossilis* sp. nov. by the general shapes of pronotum and elytra, wider mesoscutellum, paler head, and the absence of distinct elytral maculation.

Genus *Atractocerus* Palisot de Beauvois, 1802

Atractocerus sp.

(Figs. 1e, 2e, j and Supplementary Figs. 2e, 10)

Material. FMNHINS-3965992 (Supplementary Fig. 2e), a complete, relatively well, preserved female adult. Ventral side is largely not visible. Early Middle Miocene amber (ca. 16 Mya[23]), from the Dominican Republic, no further details are available.

Diagnosis. The fossil can be placed in the extant genus *Atractocerus* using the following diagnostic characters[17]: head clearly narrower than pronotum; large, contiguous eyes; short fusiform antenna; broadened mesoscutellum that occupies more than two thirds the width of the elytral base; and extremely shortened, small elytra. This fossil taxon may be distinguished from other congeners by the following characters: smaller body; strongly fusiform, but distally slender, antennae; minute, modified and elevated elytra; and the structures of the female genitalia. The ventral side is not fully visible. Further comparison with the modern congeners is difficult because of a poor visibility of the ventral side and the ambiguity of the species limit of the extant *Atractocerus*.

Description. Female. Body (Fig. 1e) medium in size, 18.25 mm long, elongate, narrowly subparallel sided, moderately dorsoventrally flattened. Colour uniformly dark brown to black; eyes, antennae, legs, and lateral areas of abdomen paler; dorsum without marking or maculation. Surface leathery, covered with fine setose punctures, each seta stout, relatively long. Head (Supplementary Fig. 10a) medium in size, 1.55 mm long and 1.61 mm wide, broadly oval, somewhat horizontally produced, slightly narrower than pronotum; basal part

of head moderately narrowed behind eyes, forming loose neck. Eyes (Supplementary Fig. 10a, d) large, conspicuous, nearly contiguous, occupying extensive parts of head surface. Antenna inserted under lateral, supra-antennal ridge. Antennae (Supplementary Fig. 10d) 11-segmented, short, strongly fusiform, slender (apical half distinctly slender), strongly narrowing anteriorly; surface setose with fine setae. Mouthparts not visible. Pronotum (Supplementary Fig. 10a) small, 1.55 mm long and 1.70 mm wide, subquadrate, weakly transverse (length/width = 0.91); lateral margins nearly straight, with slightly produced anterior margin and nearly truncate, but moderately sinuate, posterior margin; midline of pronotal disc not well visible. Mesoscutellum (Supplementary Fig. 10a) large, transverse, occupying most parts of elytral base; dorsum smooth; details not well visible. Elytra (Figs. 1e, 2e, j and Supplementary Fig. 10a) brachelytrous, extremely strongly reduced, distinctly modified, exposing most part of abdomen; each elytron minute, crescent-shaped, approximately 2.1 times longer than wide (1.37 mm long, 0.64 mm wide); dorsum irregularly elevated, with couple of longitudinal, arcuate carinae on each elytron. Hindwing (Figs. 1e, 2e) exposed, not folded beneath elytra; marginal C+Sc+R vein well developed, without radial cell; r-m crossvein (Figs. 1e, 2e) present, conspicuous; M+Cu fork absent. Legs slender and thin; procoxae thick, robust, moderately deformed; protibiae without tibial terminal spur; protarsi (Supplementary Fig. 10c) long, moderately longer than protibiae, segmentation not visible; mesotibia (Supplementary Fig. 10c) bar-like, lacking tibial terminal spur; mesotarsi (Supplementary Fig. 10b) long, slightly longer than mesotibiae, segmentation not visible; metatibia (Supplementary Fig. 10b) without tibial terminal spur; metatarsi distinctly long, much longer than metatibiae, segmentation not well visible. Abdomen (Figs. 1e, 2e and Supplementary Figs. 10e) slender, cylindrical, moderately to rather strongly flattened; abdominal segments II-VII visible dorsally; segments V and VI weakly explanate along lateral margins, while segment VII with its lateral areas much strongly broadly explanate and depressed (Figs. 1e, 2e); tergite VIII small and retracted into penultimate segment. Female genitalia (Supplementary Fig. 10e) moderately exposed, well sclerotised; coxites slender, narrowly elongate; stylus narrowly elongate, flattened, gradually narrowing apically, with setose apex.

Remarks. The distribution of *Atractocerus* in Central and South America (including the Dominican Republic), Africa, Madagascar, India, Sumatra, and northern Australia suggests Gondwanan relictualism[11]. Therefore, the occurrence of *Atractocerus* in Dominican amber is congruent with the modern congeners. Grimaldi & Engel[2] mentioned a much larger (ca. 29 mm) unnamed *Atractocerus* in Dominican amber, but the elytra were not illustrated.

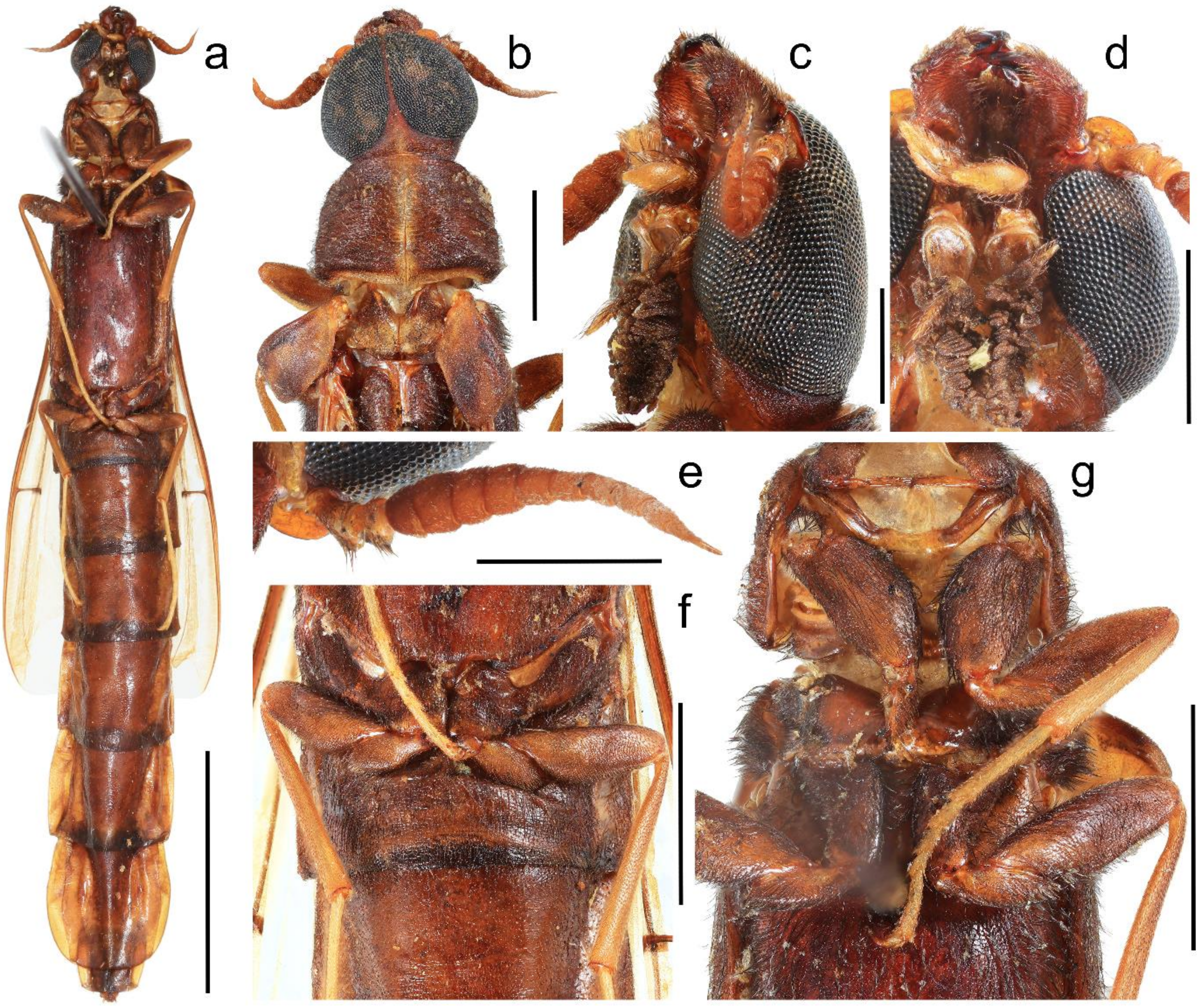

Supplementary Fig. 1 Extant atractocerine beetle, *Atractocerus brasiliensis* Lepeletier & Audinet-Serville from Bluefields, Nicaragua. **a** habitus, ventral view. **b** forebody, dorsal view. **c** head, lateral view, showing modified maxillary palp organ. **d** head partial, ventral view, showing modified maxillary palp organ. **e** antenna. **f** metacoxae, ventral view. **g** pro-and mesothorax, showing cylindrical procoxae. Scale bars: 1 cm (**a**), 3 mm (**b, f, g**), 1 mm (**c**), 1.5 mm (**d**, **e**).

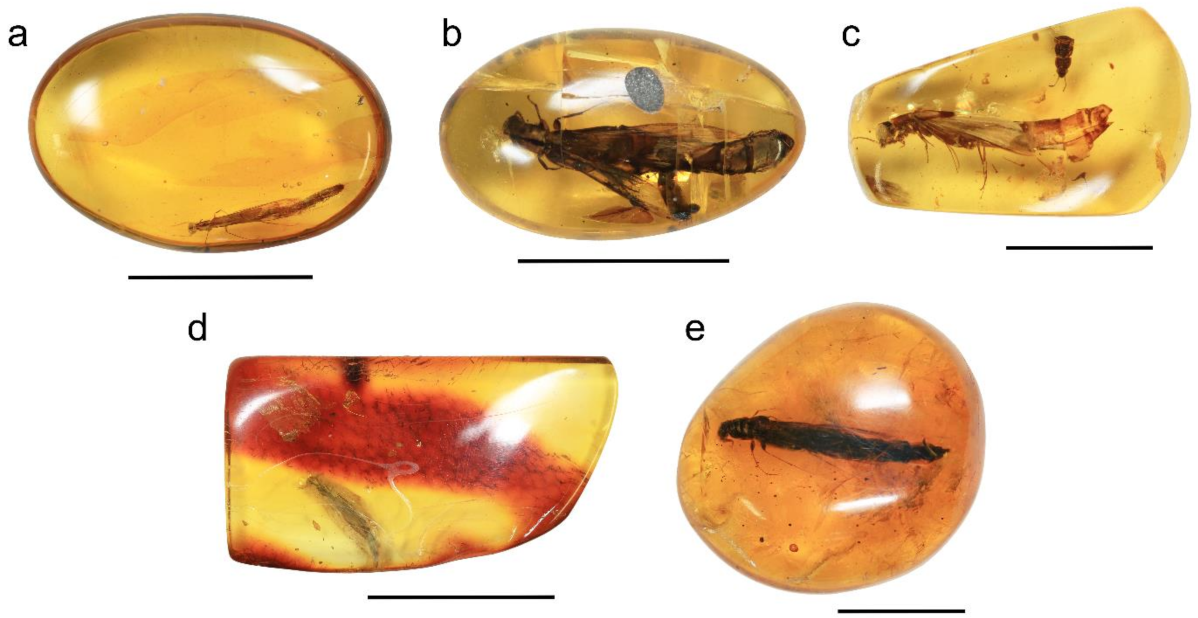

Supplementary Fig. 2 Amber specimens with atractocerine inclusions. **a**–**c** mid-Cretaceous Burmese amber, **d** mid-Eocene Baltic amber, **e** early Middle Miocene Dominican amber. (**a**) *Vetatractocerus burmiticus* gen. et sp. nov., holotype, FMNHINS-3965988. (**b**) *Raractocetus extinctus* sp. nov., holotype, FMNHINS-3965989. (**c**) *R. fossilis* sp. nov., holotype, FMNHINS-3965990. (**d**) *R. balticus* sp. nov., holotype, FMNHINS-3965991. (**e**) *Atractocerus* sp., FMNHINS-3965992. Scale bars: 1 cm (**a**, **b**, **e**), 5 mm (**c**), 2 cm (**d**).

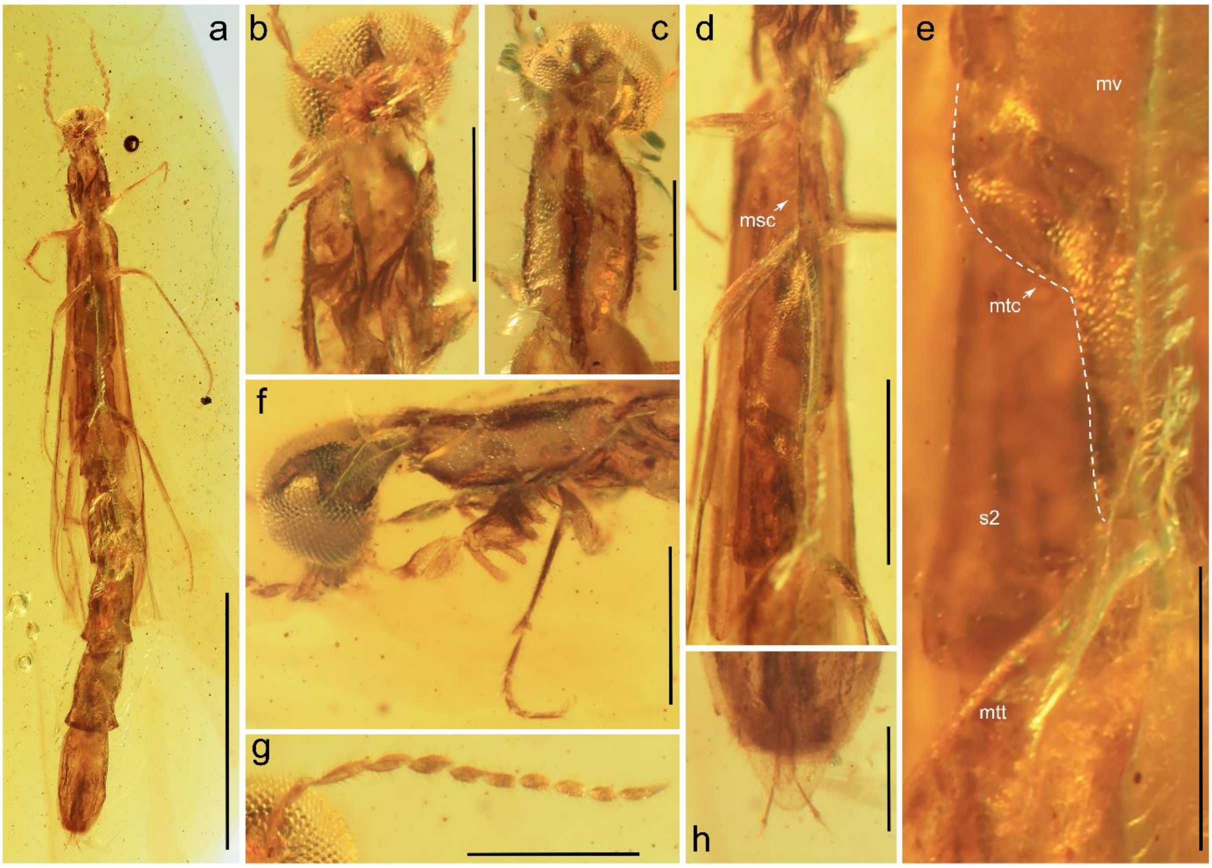

Supplementary Fig. 3 Extinct atractocerine beetle, *Vetatractocerus burmiticus* gen. et sp. nov., in mid-Cretaceous Burmese amber, holotype, FMNHINS-3965988. **a** habitus, ventral view. **b** head and prothorax, ventral view. **c** head and prothorax, dorsal view. **d** meso- and metathorax, ventral view, showing distinctly modified metacoxae. **e** enlargement of (**d**), with a whitish dashed line indicating distinctly modified metacoxae. **f** head and prothorax, lateral view. **g** antenna, right. **h** abdominal apex, ventral view, showing exposed female genitalia. Abbreviations: msc, mesocoxa; mtt, metatibia; mv, metaventrite; mtc, metacoxae; s2, sternite. Scale bars: 3 mm (**a**), 0.5 mm (**b, c, e, g**), 1 mm (**d**, **f**), 0.3 mm (**h**).

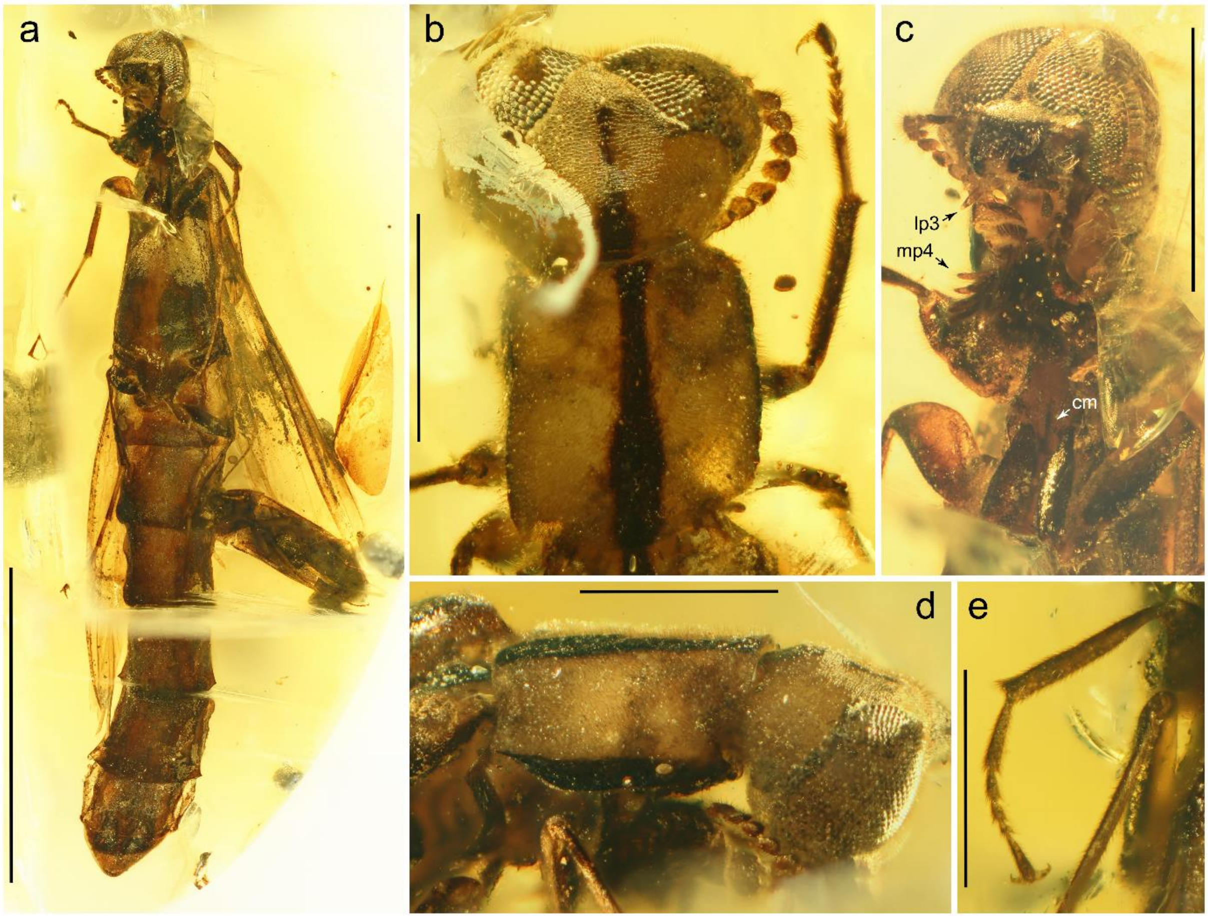

Supplementary Fig. 4 Extinct atractocerine beetle, *Raractocetus extinctus* sp. nov., in mid-Cretaceous Burmese amber, holotype, FMNHINS-3965989. **a** habitus, ventral view. **b** head and prothorax, dorsal view. **c** head and prothorax, ventrolateral view, showing modified maxillary palp organ and longitudinally carinated mesoventrite. **d** head and prothorax, lateral view. **e** protibia and protarsus, left. Abbreviations: cm, longitudinal carina on mesoventrite; lp3, labial palpomere 3; mp4, maxillary palpomere 4. Scale bars: 5 mm (**a**), 1 mm (**b, d, e**), 1.5 mm (**c**).

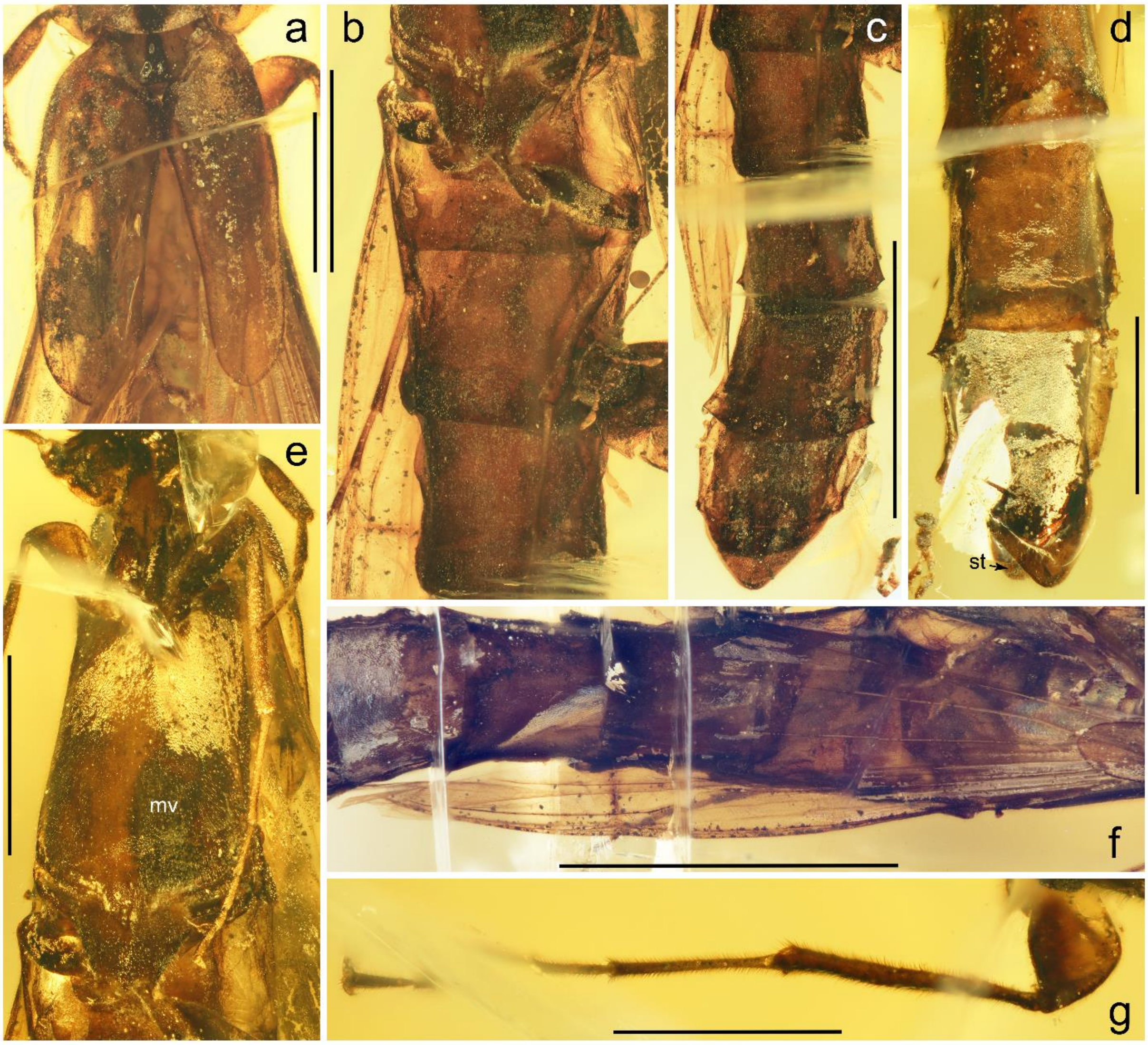

Supplementary Fig. 5 Extinct atractocerine beetle, *Raractocetus extinctus* sp. nov., in mid-Cretaceous Burmese amber, holotype, FMNHINS-3965989. **a** mesoscutellum and elytra, dorsal view. **b** metalegs, ventral view, showing moderately modified metacoxae. **c** abdomen, ventral view. **d** abdomen, dorsal view, showing barely exposed female genitalia. **e** meso- and metathorax, ventral view, showing markedly elongated metaventrite. **f** hindwing, right. **g** mesotibia and mesotarsus, right. Abbreviations: as, apex of stylus; mv, metaventrite. Scale bars: 1 mm (**a**, **g**), 1.5 mm (**b, d, e**), 3 mm (**c**, **f**).

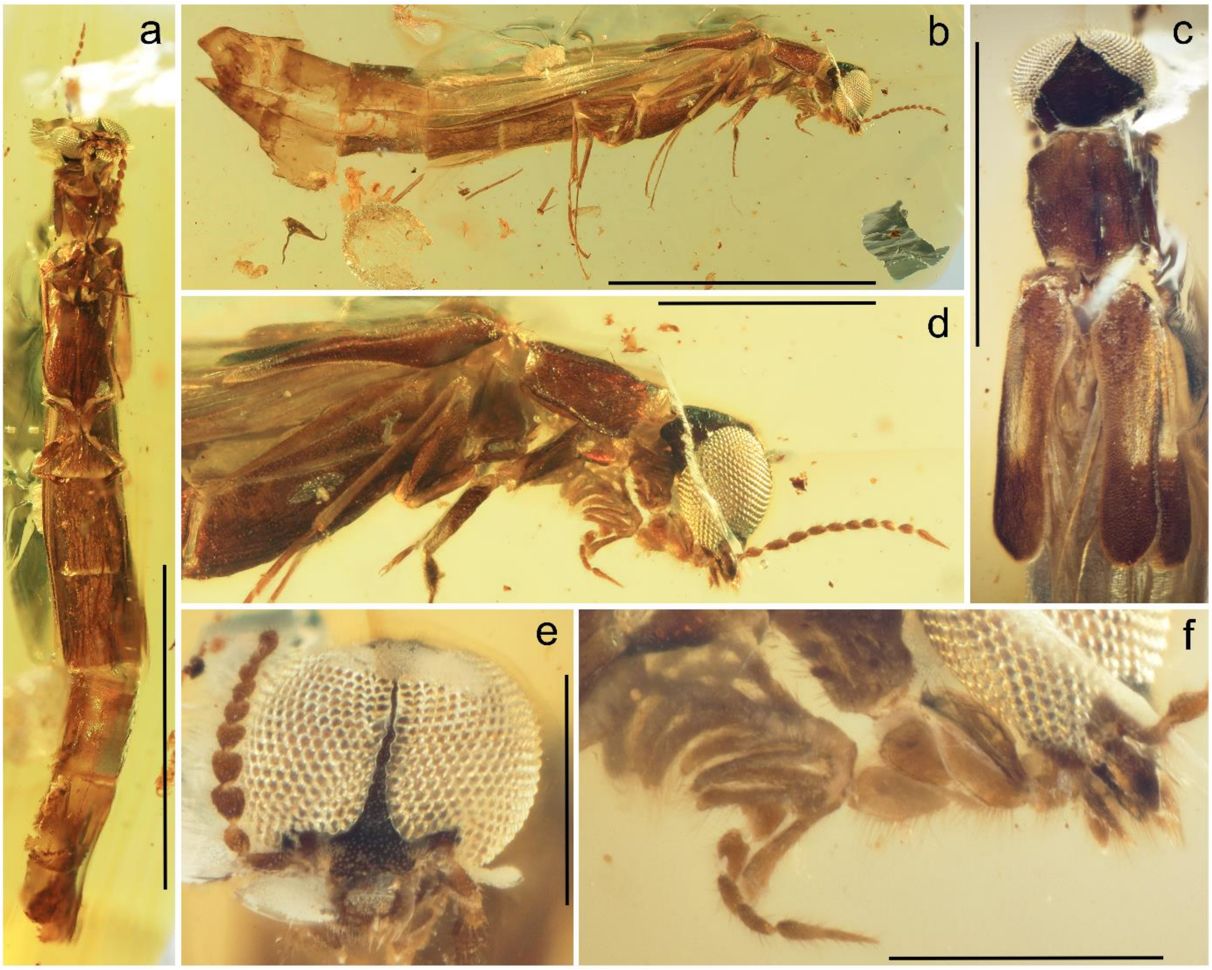

Supplementary Fig. 6 Extinct atractocerine beetle, *Raractocetus fossilis* sp. nov., in mid-Cretaceous Burmese amber, holotype, FMNHINS-3965990. **a** habitus, ventral view. **b** habitus, lateral view. **c** forebody, dorsal view. **d** forebody, lateral view. **e** head, frontal view, showing large and contiguous eyes. **f** maxillary pulpus, showing distinctly modified maxillary palpomere 4. Scale bars: 3 mm (**a**, **b**), 1.5 mm (**c**), 1 mm (**d**), 0.5 mm (**e**, **f**).

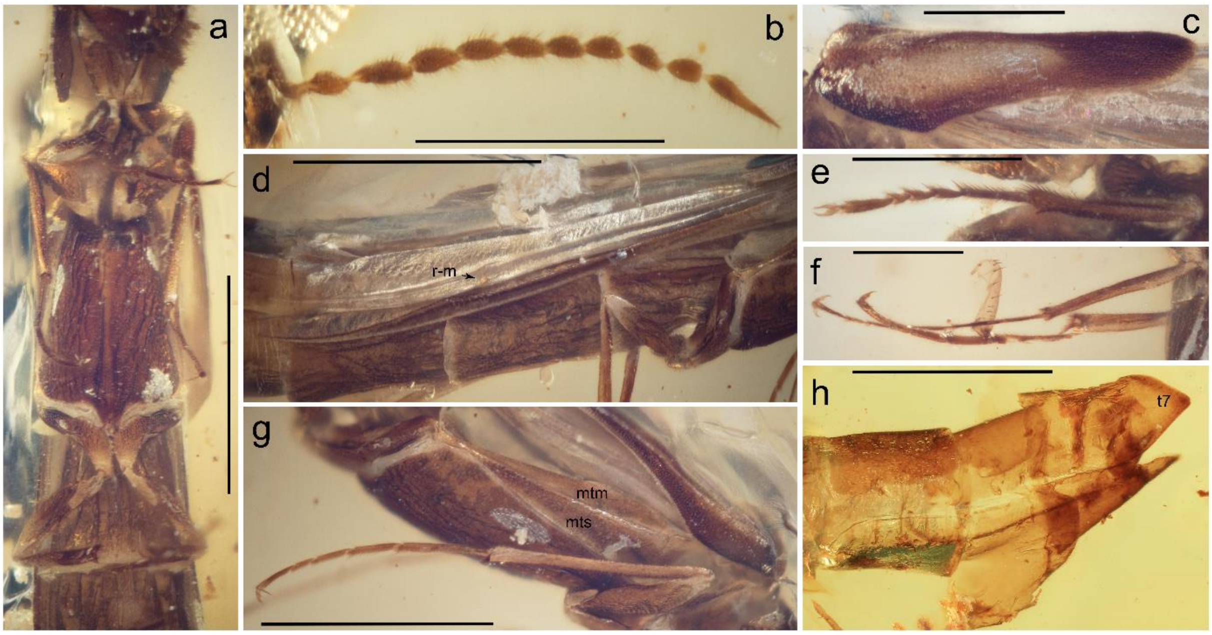

Supplementary Fig. 7 Extinct atractocerine beetle, *Raractocetus fossilis* sp. nov., in mid-Cretaceous Burmese amber, holotype, FMNHINS-3965990. **a** pro-, meso-, and metathorax, ventral view, showing moderately modified metacoxae. **b** antenna, right. **c** elytron, lateral view. **d** hindwing, right. **e** protibia and protarsus, left. **f** metalegs, lateral view. **g** mesoleg, metepisternum, and metepimeron, right. **h** abdominal apex, lateral view. Abbreviations: mtm, metepimeron; mts, metepisternum; r-m, r-m crossvein; t7, abdominal tergite 7. Scale bars: 1 mm (**a**, **g**), 0.5 mm (**b**, **c**, **e**, **f**), 1.5 mm (**d**, **h**).

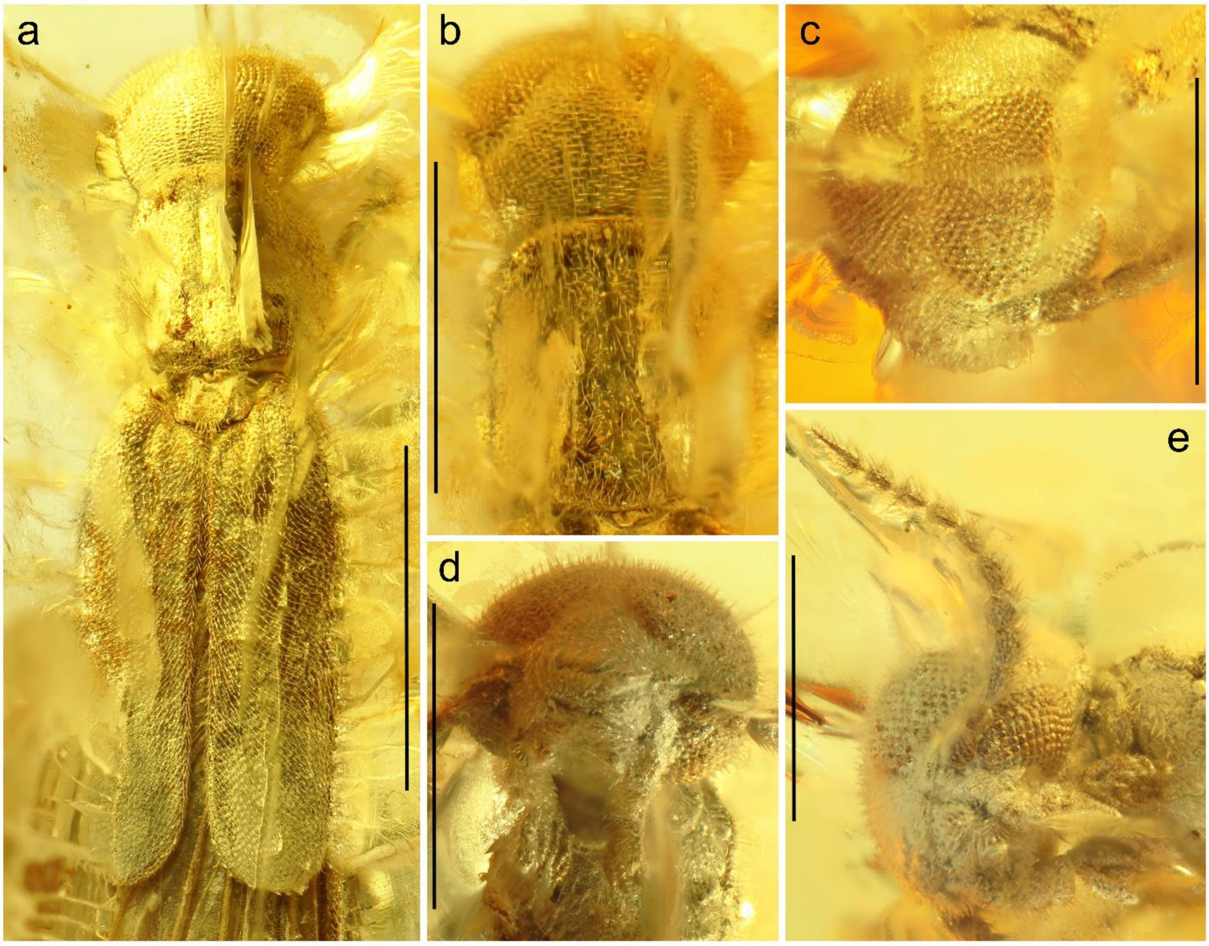

Supplementary Fig. 8 Extinct atractocerine beetle, *Raractocetus balticus* sp. nov., in mid-Eocene Baltic amber, holotype, FMNHINS-3965991. **a** forebody, dorsal view. **b** head and pronotum, dorsal view. **c** head, frontal view, showing large and contiguous eyes. **d** head and prothorax, ventral view, showing distinctly modified maxillary palpomere 4. **e** left antenna and head, ventral view, showing distinctly modified maxillary palpomere 4. Scale bars: 1.5 mm (**a**), 1 mm (**b**–**e**).

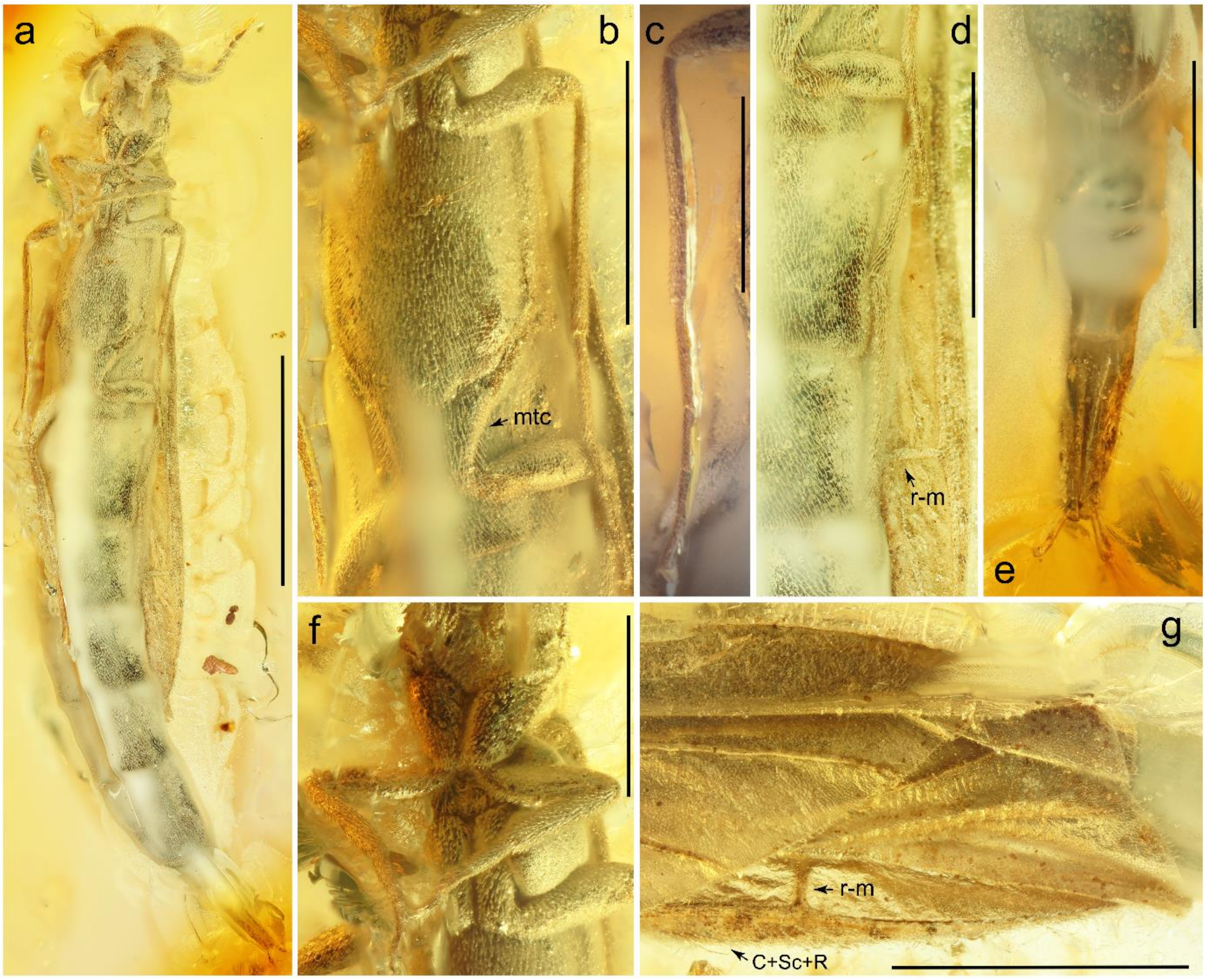

Supplementary Fig. 9 Extinct atractocerine beetle, *Raractocetus balticus* sp. nov., in mid-Eocene Baltic amber, holotype, FMNHINS-3965991. **a** habitus, ventral view. **b** metathorax, ventral view, showing moderately modified metacoxae. **c** mesoleg, right. **d** metaleg, left. **e** abdominal apex, dorsal view, showing exposed female genitalia. **f** pro-and mesothorax, showing cylindrical pro- and mesocoxae. **g** hindwing, left. Abbreviations: C+Sc+R, marginal C+Sc+R vein; mtc, metacoxae; r-m, r-m crossvein. Scale bars: 3 mm (**a**), 1.5 mm (**b, d, g**), 1 mm (**c**, **e**, **f**).

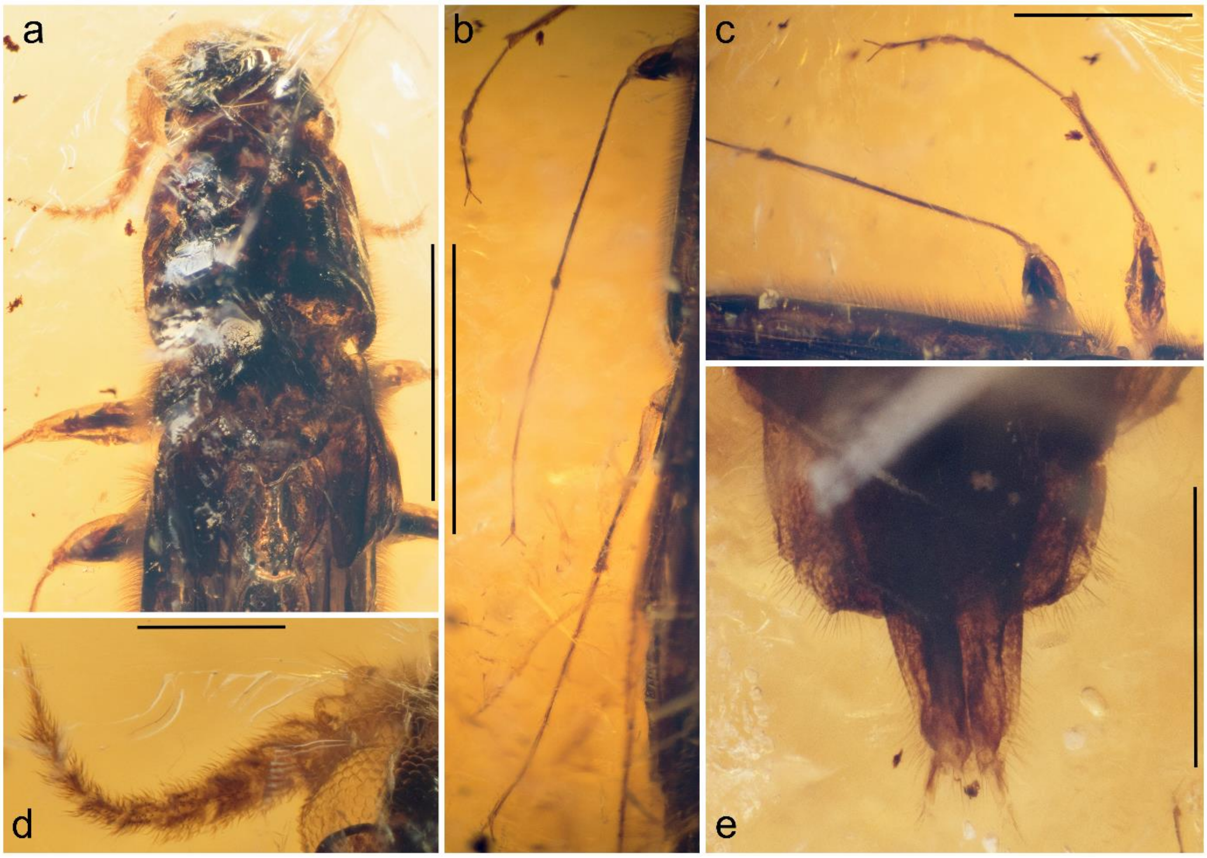

Supplementary Fig. 10 Extinct atractocerine beetle, *Atractocerus* sp., in early Middle Miocene Dominican amber, holotype, FMNHINS-3965992. **a** forebody, dorsal view. **b** legs, left. **c** proleg and mesotibia, left. **d** antenna, left. **e** abdominal apex, dorsal view, showing exposed female genitalia. Scale bars: 2 mm (**a**), 3 mm (**b**), 1.5 mm (**c**), 0.5 mm (**d**), 1 mm (**e**).
